# USP5 promotes glycolysis of fibroblast-like synoviocytes by stabilizing the METTL14/m^6^A/GLUT1 axis in rheumatoid arthritis

**DOI:** 10.1038/s41420-025-02890-2

**Published:** 2025-12-03

**Authors:** Xuan’an Li, Min Ling, Zhongchi Wen, Chonghua Jiang, Xiaohua Tan

**Affiliations:** 1 Department of Orthopaedics, Hunan Cancer Hospital/the Affiliated Cancer Hospital of Xiangya School of Medicine, Central South University, Changsha, Hunan Province P. R. China; 2 Department of Neurosurgery, Brain Hospital of Hunan Province, Changsha, Hunan Province P. R. China; 3 Department of Pathophysiology, Xiangya School of Medicine, Central South University, Changsha, Hunan Province P. R. China; 4 Department of Neurosurgery, Geriatric Hospital of Hainan, Haikou, Hainan Province P. R. China; 5 Morphological Center, Xiangya School of Basic Medical Science, Central South University, Changsha, Hunan Province P. R. China

**Keywords:** Inflammasome, Immunotherapy

## Abstract

Fibroblast-like synoviocytes (FLSs) contribute to the advancement of rheumatoid arthritis (RA) through enhanced metabolic reprogramming. This research focused on exploring the role and underlying mechanism of ubiquitin-specific protease 5 (USP5) in modulating the glycolysis and activation of RA-FLSs. Here, we identified that knockdown of USP5 in RA rats reduced synovial inflammation and glycolytic activity, as evidenced by decreased serum lactate levels and GLUT1 expression. In RA-FLSs, USP5 knockdown or treatment with 2-DG reduced cell proliferation, migration, invasion, cytokine production, and glycolysis, while increased apoptosis. Mechanistically, USP5 stabilized METTL14 by inhibiting its ubiquitination, while METTL14 enhanced the m^6^A modification of GLUT1 mRNA, thereby increasing its expression. Furthermore, overexpression of METTL14 partially reversed the effects of USP5 knockdown on glycolysis and inflammatory activation in RA-FLSs. Additionally, knockdown of METTL14 inhibited RA-FLS glycolysis and inflammatory activation by downregulating GLUT1. Collectively, USP5 stabilized METTL14-mediated m^6^A modification of GLUT1 by inhibiting the ubiquitination of METTL14, thereby enhancing glycolysis and inflammatory activation in RA-FLSs. These results suggest that the USP5/METTL14/GLUT1 axis could be a potential therapeutic target for RA.

## Highlights

- USP5 enhances glycolysis and activation of rheumatoid arthritis fibroblast-like synoviocytes via METTL14.
- METTL14 deubiquitination and stabilization by USP5 enhance the m^6^A modification of GLUT1 mRNA in rheumatoid arthritis.
- METTL14 promotes GLUT1-dependent glycolysis and activation of rheumatoid arthritis fibroblast-like synoviocytes.
- Targeting the USP5/METTL14/GLUT1 axis offers a potential therapeutic strategy for rheumatoid arthritis.

## Introduction

Rheumatoid arthritis (RA) is a chronic autoimmune disorder marked by persistent synovial inflammation and gradual joint damage, which significantly impacts patient quality of life [1, 2]. Current treatment strategies aim to control inflammation and prevent joint damage, but challenges remain in fully understanding the underlying mechanisms that drive RA pathology [3]. Fibroblast-like synoviocytes (FLSs) are key players in RA progression for their role in maintaining inflammation, degrading the extracellular matrix, and driving joint destruction [4, 5]. Targeting the activation of FLSs may thus offer new avenues for therapeutic intervention.

Glycolysis, a major metabolic pathway, is essential for the activation of RA-FLSs [6]. It has been observed that elevated glycolysis in RA synovial tissue leads to persistent inflammation and joint damage [7]. Inhibiting glycolysis can induce apoptosis of RA-FLSs, inhibit cell proliferation, and invasion [8, 9]. Targeting specific glycolytic enzymes or intermediates in the synovium may have therapeutic effects on RA. Glucose transporter 1 (GLUT1), a critical protein for glucose uptake, is significantly upregulated in synovial samples, including FLSs from RA patients [10]. GLUT1 has been implicated in promoting inflammatory functions, cell proliferative, and invasive properties in RA-FLSs [11]. By understanding the mechanisms that regulate glycolysis and GLUT1 expression in RA-FLSs, novel targets for disrupting the inflammatory and destructive potential of these cells can be identified.

Recent studies have highlighted the importance of N6-methyladenosine (m^6^A) modification in regulating gene expression in various diseases, including RA [12–14]. m^6^A modification, an internal RNA modification in eukaryotic cells, involves the methylation of the nitrogen at the sixth position of adenosine, influencing RNA stability, splicing, translation, and degradation [15, 16]. Among the key components of the m^6^A machinery, methyltransferase-like 14 (METTL14), an m^6^A methyltransferase, is overexpressed in RA synovial tissues and is essential for regulating immunoinflammatory markers and clinical symptoms [17]. Our previous research has also demonstrated that METTL14 exhibits elevated expression in RA and promotes the activation of RA-FLSs, suggesting its significant involvement in RA pathogenesis [18]. However, the specific mechanisms through which METTL14 regulates glycolysis in RA-FLSs, and how it contributes to disease progression, remain to be elucidated.

Ubiquitin-specific protease 5 (USP5), known for its deubiquitinating activity, plays a significant role in regulating proinflammatory functions [19]. Elevated expression of USP5 have been detected in RA synovial tissues, where it promotes inflammatory responses in RA-FLSs [20]. Evidence suggests that USP5 may regulate key signaling molecules involved in FLS activation, yet its role in metabolic regulation, specifically glycolysis, has not been explored. It was reported that ubiquitin-specific proteases can mediate the deubiquitination of METTL14 protein [21]. Furthermore, the m^6^A modification of GLUT1 mRNA is closely associated with glucose metabolism [22]. Therefore, in this study, we propose that USP5 stabilizes METTL14 by preventing its ubiquitination, thereby enhancing the m^6^A modification of GLUT1 mRNA and promoting glycolysis in RA-FLSs. We will investigate the USP5-METTL14-GLUT1 regulatory axis and its role in the activation of RA-FLSs. By elucidating these interactions, we aim to offer advanced insights into the metabolic regulation of RA-FLSs and to identify innovative therapeutic targets for RA treatment.

## Results

### USP5 promoted glycolysis and the progression of RA

**Fig. 1:**
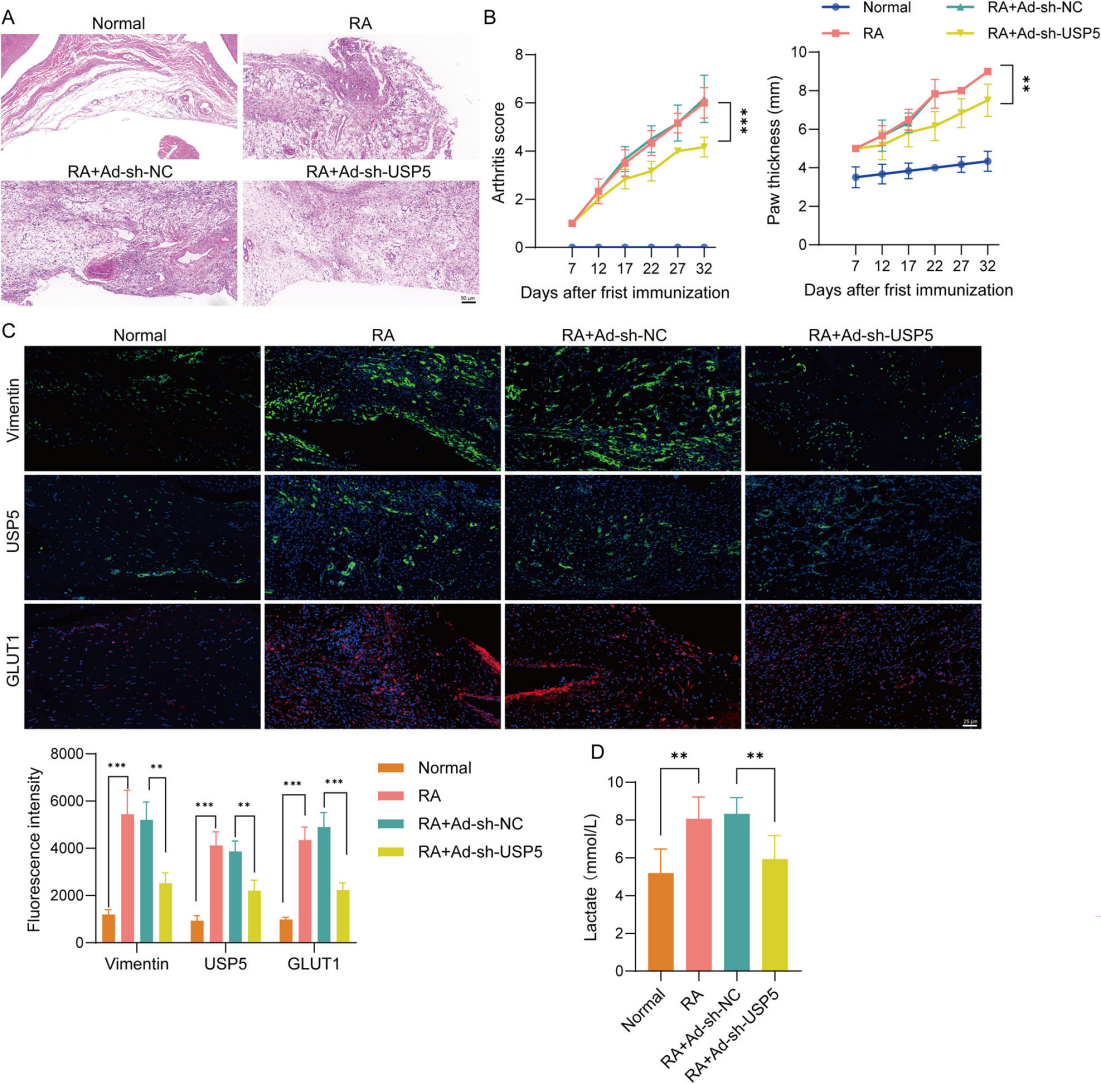
USP5 promoted glycolysis and the progression of RA. **A** H&E staining assessed synovial tissue pathological changes among the normal group, RA model group, and USP5 knockdown groups. **B** Arthritis scores and paw thickness were evaluated. **C** Immunofluorescence was used to detect Vimentin, USP5, and GLUT1 expression in synovial tissues. **D** Serum lactate levels were measured using a lactate assay kit to compare metabolic activity among the different groups. *N* = 6. **p < 0.01, ***p < 0.001.

To investigate the specific function of USP5 in RA progression, a rat model of RA was initially developed, and synovial inflammation was evaluated. Histopathological analysis using H&E staining was conducted to evaluate synovial changes, revealing that the RA group exhibited significant synovial hyperplasia and inflammatory cell infiltration, thus confirming the successful establishment of the RA rat model. The knockdown of USP5 led to marked improvement in synovial hyperplasia and a reduction in inflammatory cell infiltration (Fig. 1A). Arthritis scores and paw thickness were measured to further assess the effect of USP5 on RA severity, revealing that USP5 knockdown significantly reduced arthritis severity compared to the RA model group (Fig. 1B). Immunofluorescence analysis further determined the effect of USP5 on glycolysis and its potential involvement in RA pathogenesis. Vimentin, USP5, and GLUT1 expressions were markedly increased in the RA group, indicating increased glycolytic activity and synovial activation. Knockdown of USP5 led to a substantial decrease in Vimentin and GLUT1 expression (Fig. 1C). To assess the glycolytic activity associated with USP5, serum lactate levels were measured. RA rats exhibited significantly elevated serum lactate levels compared to the normal group, while USP5 knockdown decreased lactate levels (Fig. 1D). These findings indicate that USP5 enhances glycolysis in RA, potentially contributing to synovial inflammation. To confirm the clinical relevance of our findings, Western blot analysis was performed using human synovial tissues from RA patients and normal controls. The results showed that USP5, METTL14, and GLUT1 were significantly upregulated in RA tissues (Supplementary Fig. S1A).

### USP5 promoted the activation of RA-FLSs by regulating glycolysis

**Fig. 2:**
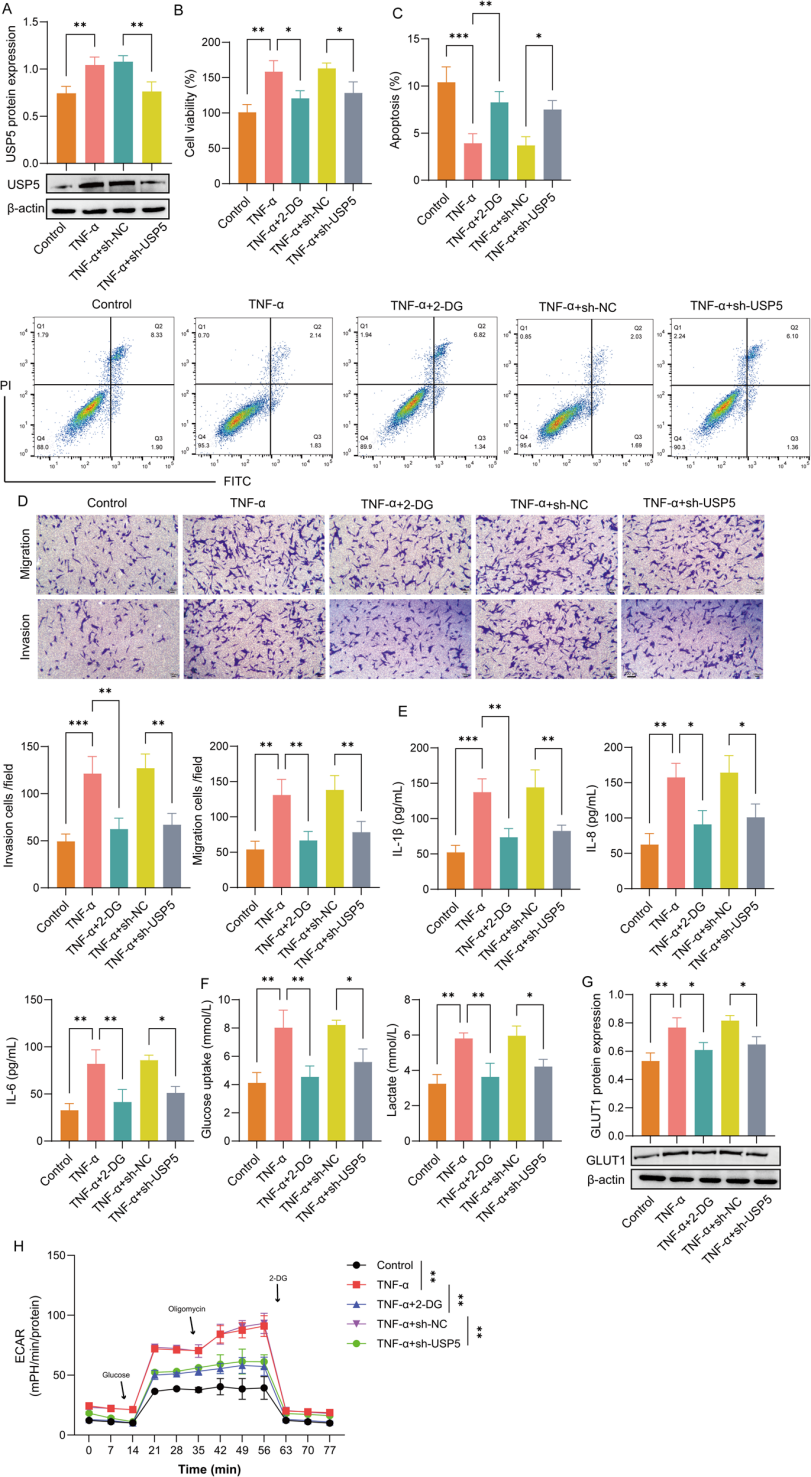
USP5 promoted glycolysis and activation of RA-FLSs. **A** Western blot analysis of USP5 expression in TNF-α-treated RA-FLSs, with and without USP5 knockdown. **B** CCK-8 assays for cell proliferation across the TNF-α-treated, USP5 knockdown, and 2-DG-treated groups. **C** Flow cytometry analysis for apoptosis rate in RA-FLSs under different treatment conditions. **D** Transwell assays for cell migration and invasion. **E** ELISA for inflammatory cytokine levels (IL-1β, IL-6, IL-8) in different treatment groups. **F** Glucose uptake and lactate production in RA-FLSs were assessed using corresponding kits. **G** Western blot analysis of GLUT1 expression in different groups. **H** ECAR analysis for glycolytic capacity across different treatment groups. *N* = 3. *p < 0.05, **p < 0.01, ***p < 0.001.

To explore the particular function of USP5 in activating RA-FLSs, we treated RA-FLSs with TNF-α to mimic the inflammatory environment of RA. Western blot analysis revealed a significant upregulation of USP5 expression in RA-FLSs following TNF-α treatment. Conversely, USP5 knockdown led to a notable decrease in its level (Fig. 2A). The CCK-8 results indicated that TNF-α treatment significantly promoted RA-FLS proliferation, whereas knockdown of USP5 or treatment with 2-DG (an inhibitor of glycolysis) inhibited this effect (Fig. 2B). Flow cytometry analysis revealed that TNF-α treatment significantly decreased apoptosis in RA-FLSs, while USP5 knockdown or 2-DG treatment led to a significant increase in apoptosis (Fig. 2C). Transwell migration and invasion assays demonstrated that TNF-α significantly promoted cell migration and invasion, whereas USP5 knockdown or 2-DG treatment suppressed these effects (Fig. 2D). The ELISA demonstrated that TNF-α treatment significantly increased the levels of IL-1β, IL-6, and IL-8, while USP5 knockdown or 2-DG treatment reduced their levels (Fig. 2E). Glucose uptake and lactate production measurements revealed that TNF-α treatment significantly enhanced glycolysis in RA-FLSs, whereas USP5 knockdown or 2-DG treatment markedly reduced these glycolytic parameters (Fig. 2F). Western blot analysis further showed that TNF-α treatment upregulated GLUT1 expression, which was significantly downregulated following USP5 knockdown or 2-DG treatment (Fig. 2G). Finally, ECAR analysis was used to evaluate glycolytic capacity, showing that TNF-α enhanced glycolysis in RA-FLSs, while USP5 knockdown or 2-DG treatment inhibited this glycolytic activity (Fig. 2H). Moreover, to exclude off-target effects of sh-USP5, a rescue experiment was performed in RA-FLSs using a target gene expression vector containing silent mutations (altering the base sequence of the shRNA binding site without changing the amino acid coding). Co-transfection of sh-USP5 with Rescue-USP5 restored GLUT1 expression, cell proliferation, and lactate levels (Fig. S2), confirming the specificity of sh-USP5. Collectively, these findings demonstrate that USP5 is crucial in driving the activation of RA-FLSs in an inflammatory environment by enhancing glycolysis.

### USP5 stabilized METTL14 by inhibiting its ubiquitination

**Fig. 3:**
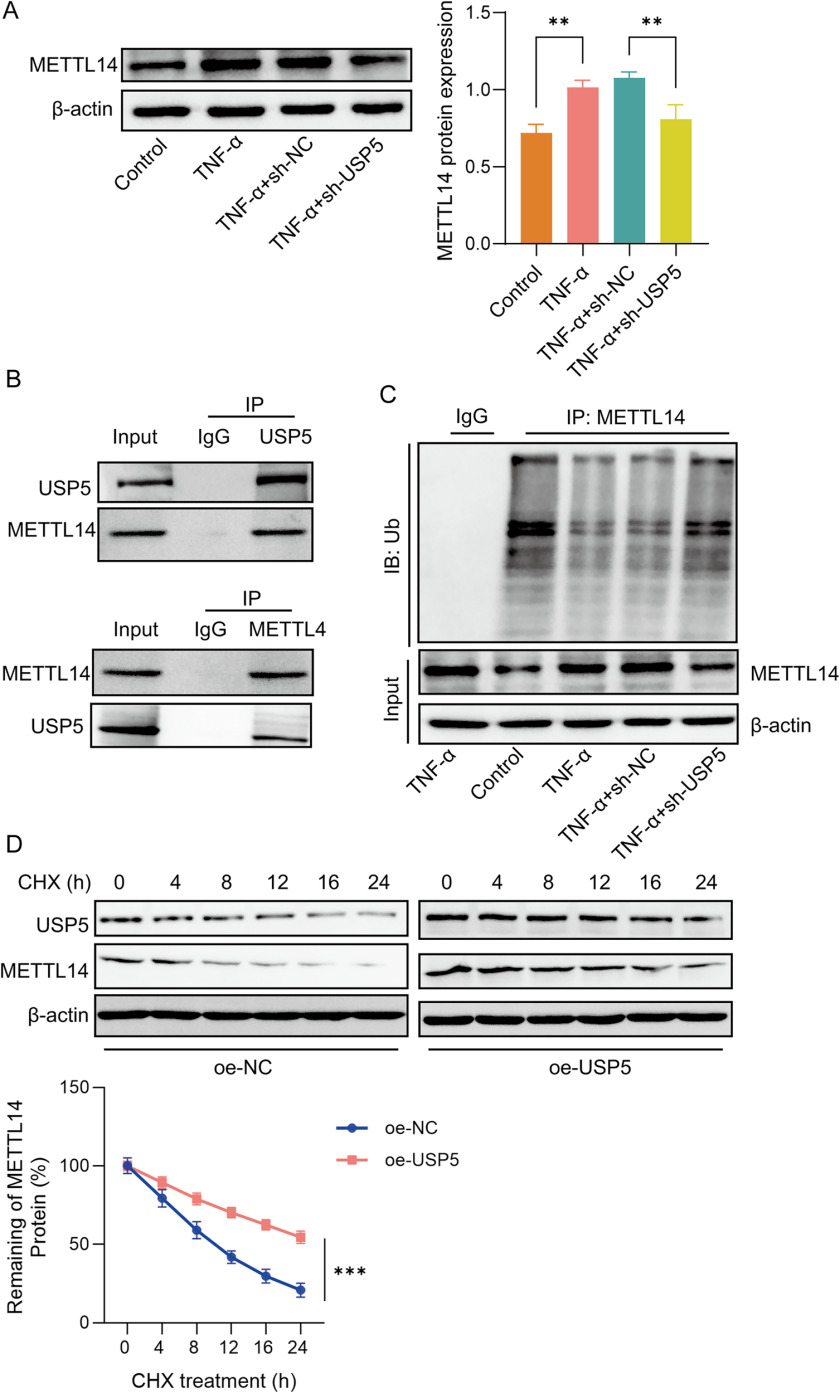
USP5 stabilized METTL14 by inhibiting its ubiquitination. **A** Western blot analysis of METTL14 expression under TNF-α treatment and USP5 knockdown conditions. **B** Co-immunoprecipitation to confirm the interaction between USP5 and METTL14 in RA-FLSs. **C** Ubiquitination assays of METTL14 under TNF-α treatment and USP5 knockdown conditions. **D** CHX chase assay showed the effect of USP5 overexpression on METTL14 protein stability in RA-FLSs. *N* = 3. **p < 0.01, ***p < 0.001.

Based on previous research regarding METTL14’s involvement in RA-FLS activation, we further investigate the relationship between USP5 and METTL14. Western blot analysis demonstrated that TNF-α treatment significantly upregulated METTL14 expression in RA-FLSs, whereas USP5 knockdown decreased METTL14 levels (Fig. 3A). Co-IP analysis confirmed that USP5 interacted directly with METTL14 in RA-FLSs (Fig. 3B). The ubiquitination assays were then conducted to determine whether USP5 affects METTL14 stability through ubiquitination. The results indicated that TNF-α treatment led to a reduction in the ubiquitination of METTL14, whereas USP5 knockdown led to increased METTL14 ubiquitination (Fig. 3C). To further confirm the role of USP5 in regulating METTL14 protein stability, a CHX chase assay was performed. The results showed that METTL14 protein degradation was significantly delayed in USP5-overexpressing RA-FLSs compared with control cells, indicating that USP5 enhances the stability of METTL14 (Fig. 3D). These findings suggest that USP5 stabilizes METTL14 by inhibiting its ubiquitination.

### USP5 regulated glycolysis and activation of RA-FLSs via METTL14

**Fig. 4:**
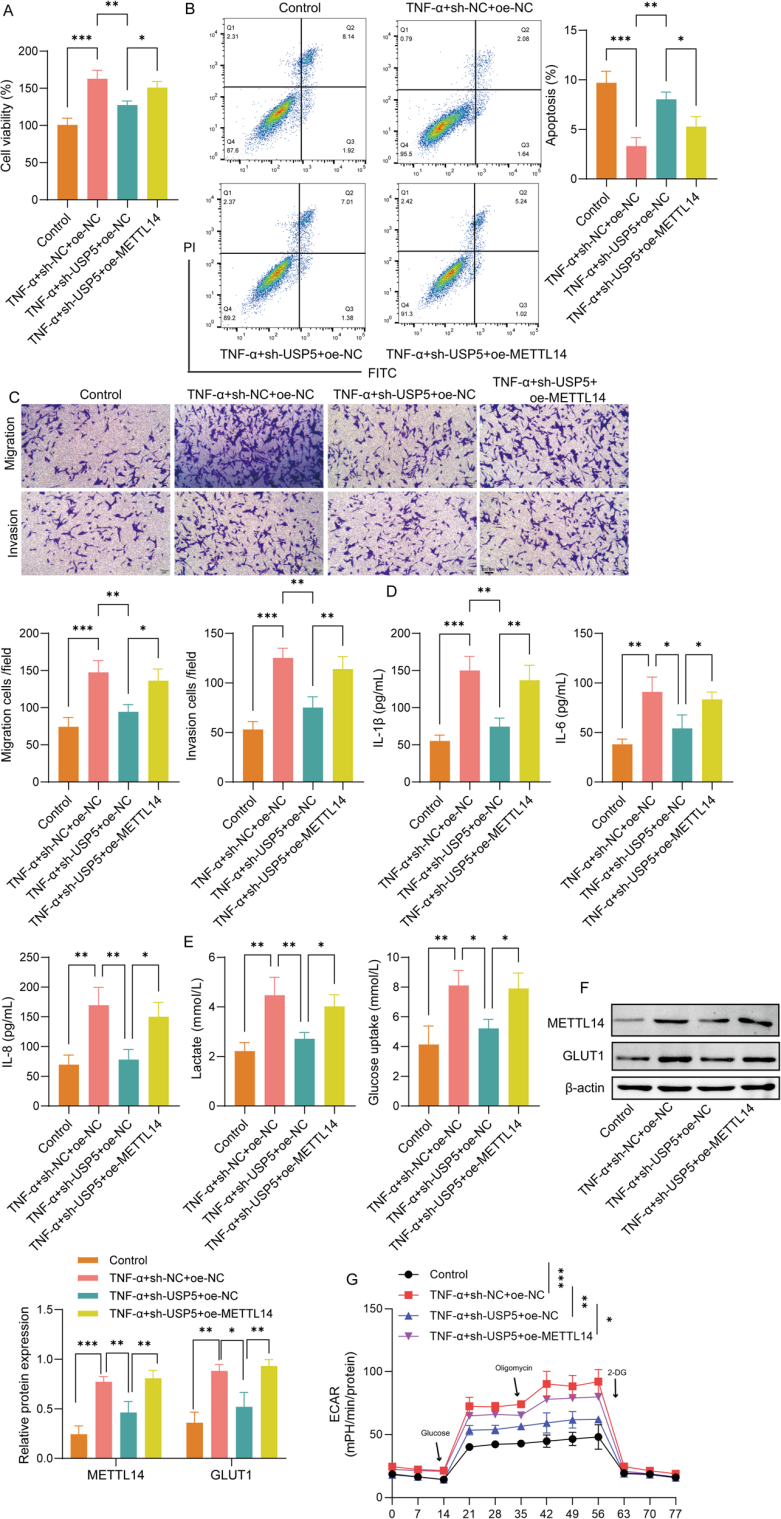
USP5 regulated glycolysis and activation of RA-FLSs via METTL14. **A** CCK-8 assays for cell viability in TNF-α-treated RA-FLSs with USP5 knockdown and METTL14 overexpression. **B** Flow cytometry analysis for apoptosis rate in different treatment groups. **C** Transwell assays for cell migration and invasion. **D** ELISA for cytokine levels (IL-1β, IL-6, IL-8) across different groups. **E** Glucose uptake and lactate production in RA-FLSs were determined using corresponding kits. **F** Western blot analysis of METTL14 and GLUT1 expression. **G** ECAR analysis for glycolysis in RA-FLSs under various treatment conditions. *N* = 3. *p < 0.05, **p < 0.01, ***p < 0.001.

To investigate the role of USP5/METTL14 axis in glycolysis and RA-FLS activation, CCK-8 assays were performed, which showed that overexpression of METTL14 partially reversed the decrease in cell viability caused by USP5 knockdown in TNF-α-treated RA-FLSs (Fig. 4A). Flow cytometry analysis demonstrated that METTL14 overexpression significantly reduced the apoptosis rate that was induced by USP5 knockdown (Fig. 4B). Transwell assays revealed that METTL14 overexpression partially counteracted the suppression of cell migration and invasion caused by USP5 knockdown (Fig. 4C). ELISA results indicated that METTL14 overexpression restored the production of IL-1β, IL-6, and IL-8 that had been decreased by USP5 knockdown (Fig. 4D). Overexpression of METTL14 upregulated the USP5 knockdown downregulated glucose uptake and lactate levels in TNF-α-induced cells (Fig. 4E). In addition, overexpression of METTL14 elevated the protein levels of METTL14 and GLUT1 that downregulated by USP5 silencing (Fig. 4F). The effect of USP5 silencing on glycolysis capacity was also inhibited by METTL14 overexpression (Fig. 4G). These findings indicate that METTL14 is essential in mediating the effects of USP5 on glycolysis and inflammatory activation in RA-FLSs.

### METTL14 promoted GLUT1 expression via the m^6^A modification of GLUT1 mRNA

**Fig. 5:**
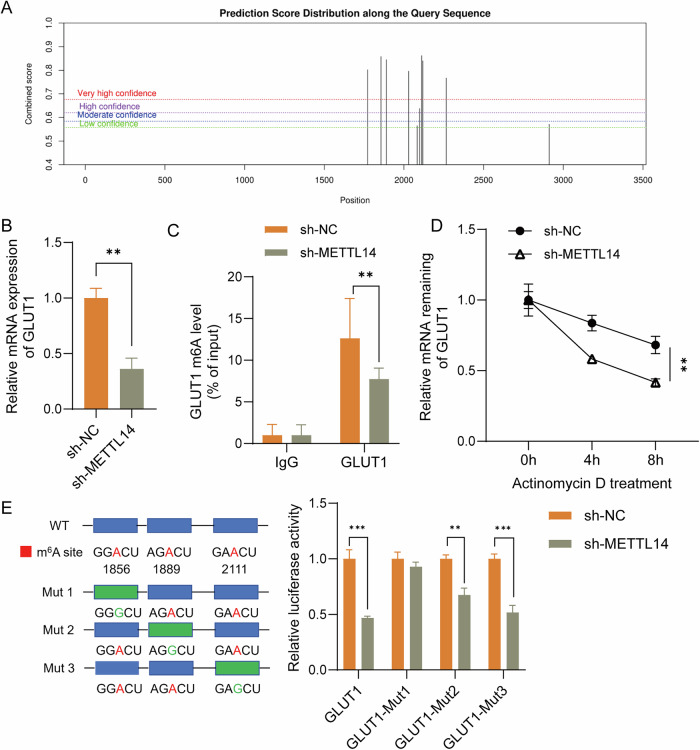
METTL14 promoted GLUT1 expression via the m^6^A modification of GLUT1 mRNA. **A** The m^6^A modification of GLUT1 mRNA was predicted using SRAMP software. **B** qPCR analysis of GLUT1 mRNA expression in RA-FLSs with METTL14 knockdown. **C** MeRIP analysis for m^6^A modification level of GLUT1 mRNA in RA-FLSs. **D** qPCR analysis for GLUT1 mRNA stability under METTL14 knockdown conditions. **E** Luciferase reporter assay of wild-type and site-directed mutant GLUT1 constructs at the predicted m⁶A sites (1856, 1889, and 2111) in RA-FLSs with METTL14 knockdown. *N* = 3. **p < 0.01, ***p < 0.001.

SRAMP (http://www.cuilab.cn/sramp/) analysis predicted that GLUT1 could be a potential target of METTL14 (Fig. 5A). To determine the effect of METTL14 on GLUT1 expression, qPCR analysis was performed, which showed that METTL14 knockdown significantly downregulated GLUT1 mRNA expression (Fig. 5B). To understand how METTL14 regulates GLUT1 mRNA, MeRIP analysis was conducted. Further analysis confirmed that METTL14 knockdown significantly decreased the m^6^A modification of GLUT1 mRNA (Fig. 5C). qPCR analysis also revealed that METTL14 knockdown lowered the stability of GLUT1 mRNA (Fig. 5D). Furthermore, site-directed mutagenesis of the predicted m⁶A sites (1856, 1889, and 2111) in GLUT1 mRNA confirmed their functional significance. METTL14 knockdown significantly reduced the luciferase activity of the wild-type GLUT1 luciferase reporter, as well as GLUT1 Mut2 and Mut3, but not GLUT1 Mut1 (Fig. 5E). This suggests that the binding between METTL14 and GLUT1 was dependent on the m⁶A modification site of Mut1. These findings indicate that METTL14 enhances the stability and expression of GLUT1 mRNA via m^6^A modification.

### METTL14 regulated GLUT1-dependent glycolysis and activation of RA-FLSs

**Fig. 6:**
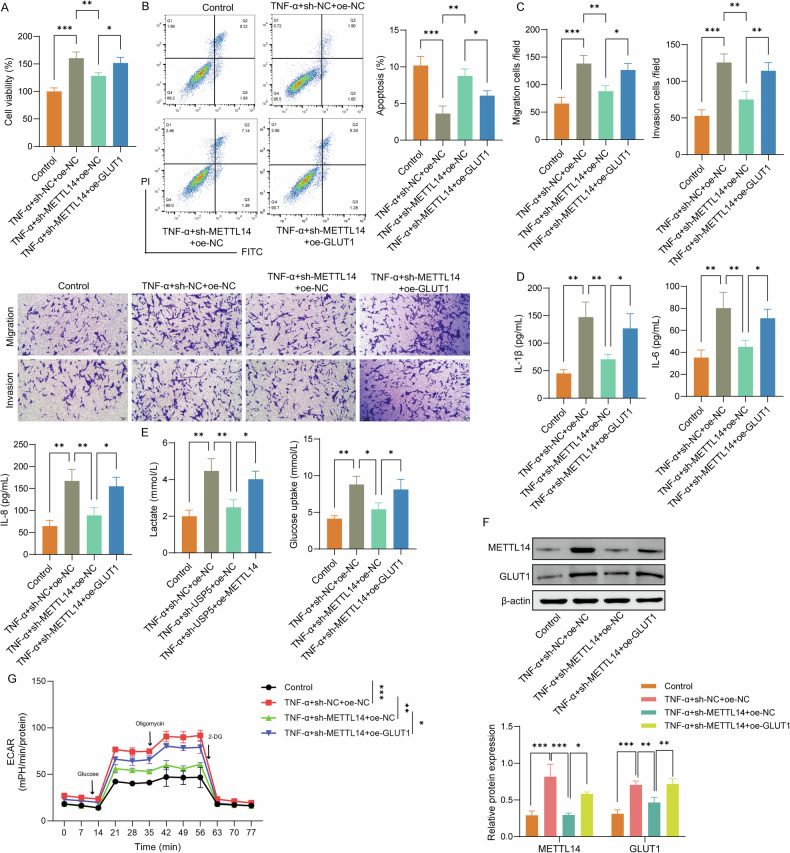
METTL14 regulated GLUT1-dependent glycolysis and activation of RA-FLSs. **A** CCK-8 assays for cell viability in TNF-α-treated RA-FLSs with METTL14 knockdown and GLUT1 overexpression. **B** Flow cytometry analysis for apoptosis rate in different groups. **C** Transwell assays for cell migration and invasion. **D** ELISA for cytokine levels (IL-1β, IL-6, IL-8) in different treatment groups. **E** Glucose uptake and lactate production in RA-FLSs with METTL14 knockdown and GLUT1 overexpression. **F** Western blot analysis of GLUT1 expression in different groups. **G** ECAR analysis for glycolytic capacity in RA-FLSs under various treatment conditions. *N* = 3. *p < 0.05, **p < 0.01, ***p < 0.001.

CCK-8 assay results showed that METTL14 knockdown markedly decreased cell viability in TNF-α-stimulated RA-FLSs. GLUT1 overexpression partially reversed this effect, restoring cell viability (Fig. 6A). Flow cytometry analysis demonstrated that METTL14 knockdown significantly increased apoptosis, whereas GLUT1 overexpression partially reversed this increase (Fig. 6B). Transwell assays were performed to evaluate cell migration and invasion, showing that METTL14 knockdown significantly reduced these abilities, while GLUT1 overexpression partially restored them (Fig. 6C). ELISA results showed that METTL14 knockdown significantly decreased the levels of IL-1β, IL-6, and IL-8, which were partially restored by GLUT1 overexpression (Fig. 6D). In addition, sh-METTL14 decreased glucose uptake and lactate production, effects that were mitigated by GLUT1 upregulation (Fig. 6E). Knockdown of METTL14 significantly downregulated TNF-α-increased GLUT1 levels and glycolysis capacity, while overexpression of GLUT1 partially reversed the inhibitory effects of sh-METTL14 on GLUT1 levels and glycolysis capacity (Fig. 6F and G). These results indicate that METTL14 promotes GLUT1-dependent glycolysis and activation of RA-FLSs.

## Discussion

RA is a long-term autoimmune disease marked by joint inflammation, cartilage and bone damage, and systemic issues [2]. The activation of FLSs is critical to the advancement of RA, largely due to their aggressive proliferation and their ability to produce significant amounts of pro-inflammatory mediators [5]. Metabolic reprogramming, particularly glycolysis, has emerged as a critical driver of FLS activation and synovial inflammation [23, 24]. In this research, we explored the function of USP5 in controlling glycolysis and inflammatory activation in RA-FLSs.

Our findings showed that USP5 significantly upregulated glycolysis and inflammatory activation in RA-FLSs, as demonstrated by increased glucose uptake, lactate production, and pro-inflammatory cytokine expression. The influence of USP5 on inflammation processes in RA-FLSs highlights its potential as a therapeutic target for RA treatment [20]. USP5 knockdown in RA rat models and cultured RA-FLSs led to decreased glycolysis, reduced inflammatory mediator production, and inhibited FLS proliferation. This aligns with findings by Bustamante et al. [25], who demonstrated that glycolysis in RA-FLSs not only supported their energy demands but also drived their invasive and inflammatory behaviors, sustaining a pathological environment within the synovium. Similarly, Zuo et al. [6] showed that glycolytic activation in RA-FLSs contributed to joint damage and inflammation by supplying the metabolic fuel needed for the pathogenic functions of these cells. By using TNF-α treatment to mimic inflammatory conditions of RA, our study confirmed that USP5 amplified glycolytic activity, suggesting its role as a metabolic activator in RA-FLSs. Moreover, our experiments confirmed that other cytokines, such as IL-1β and IL-17, could also induce USP5, METTL14, and GLUT1 expression, but TNF-α exerted the strongest effect (Fig. S1B). Consistent with previous studies that frequently employed TNF-α to induce RA-FLS activation [26], we therefore selected TNF-α for subsequent mechanistic experiments. These findings reinforce the potential of targeting metabolic pathways, such as glycolysis, to mitigate RA pathogenesis [27, 28]. Specifically, USP5 appears to be a key mediator in this process, and its inhibition may disrupt the energy production that sustains the inflammatory phenotype of RA-FLSs.

Our study further elucidated the mechanism through which USP5 stabilized METTL14 by inhibiting its ubiquitination, thereby preventing its degradation and maintaining its function in mediating m^6^A modifications. This mechanism aligns with findings by Gao et al. [29], who showed that USP family deubiquitinating enzymes contributed to the stabilization of key regulatory proteins via similar pathways in cancer cells, promoting cell proliferation and invasion. Luo et al. [20] demonstrated that USP5 deubiquitinated tumor necrosis factor receptor-associated factor 6 (TRAF6), therefore stabilizing TRAF6 to regulate the inflammatory processes of RA-FLSs. Our findings indicated that in RA, USP5 regulated METTL14 stability, which, in turn, allowed sustained m^6^A modification of mRNAs critical for glycolysis, proliferation, migration, invasion, and inflammation in FLSs. This echoes findings by Su et al. [30], who reported that METTL14 and METTL3 were upregulated in RA and that the m^6^A modification activity contributed to theses phenotypes of RA-FLSs. By revealing USP5 as a stabilizer of METTL14, our study highlights a novel layer of post-translational regulation in RA-FLSs. The preservation of METTL14 function through deubiquitination emphasizes USP5 as a master regulator of protein stability in RA, adding to the potential for USP5 to act as a treatment target for RA by modulating protein ubiquitination and glycolysis within the synovium.

GLUT1 is a central glucose transporter for glycolysis, and its overexpression in RA synovial tissues is essential for meeting the high metabolic demands of proliferative and inflammatory cells in RA [31]. There are some m^6^A site in the GLUT1 mRNA, which can be recognized by m^6^A methyltransferase to regulate its mRNA stability [32]. Our results showed that METTL14 enhanced GLUT1 mRNA stability through m^6^A modifications, promoting its expression and glycolytic flux in RA-FLSs. Previous study similarly found that METTL14 overexpression in inflammatory tissues supported the pathogenic activity of cells [18]. Furthermore, GLUT1 was specifically upregulated in RA-FLSs and was linked to increased glucose metabolism and baseline functions of RA-FLSs [11]. Given that glycolysis is a key driver of FLS proliferation and joint inflammation, targeting the m^6^A modification of GLUT1 could offer a novel approach to disrupt the metabolic and inflammatory processes in RA synovium. These results add to the accumulating evidence indicating that m^6^A modifications are integral to metabolic reprogramming in inflammatory diseases [33].

Despite these findings, our study has limitations. Primarily, our experiments were conducted in vitro and in RA rat models; thus, additional studies are required to confirm these results in human RA patients. Clinical studies are crucial to determine if the USP5/METTL14/GLUT1 axis functions similarly in human RA and whether targeted therapies can effectively reduce RA symptoms in patients. Additionally, although this study focuses on RA-FLSs, the broader impact of USP5-mediated metabolic reprogramming on immune cell interactions within the synovium remains unclear. Future research should explore how these metabolic changes affect the inflammatory milieu in the RA synovium, potentially involving other immune cells types, including macrophages and T lymphocytes. Finally, while we focused primarily on glycolysis through GLUT1, metabolic reprogramming in RA involves multiple pathways and enzymes. Other key glycolytic enzymes, including HK2, PKM2, and LDHA, may also be regulated by the USP5/METTL14 axis [34, 35]. In addition, USP5 has been reported to stabilize glycolytic enzymes such as PFKFB4 and PFKP in cancer, raising the possibility that similar mechanisms may exist in RA [36, 37]. Beyond these enzymes, glycolysis in RA is also influenced by regulators such as HIF-1α and mTOR, which integrate hypoxic and nutrient signals. USP5/METTL14 signaling may interact with these pathways to modulate glycolytic activity in RA-FLSs [26, 38]. The post-translational modifications, including phosphorylation and acetylation, may cooperate with ubiquitination in regulating protein stability and enzyme function [39, 40]. Although not addressed in our study, the potential crosstalk among these regulatory mechanisms deserves further investigation.

## Conclusions

**Fig. 7:**
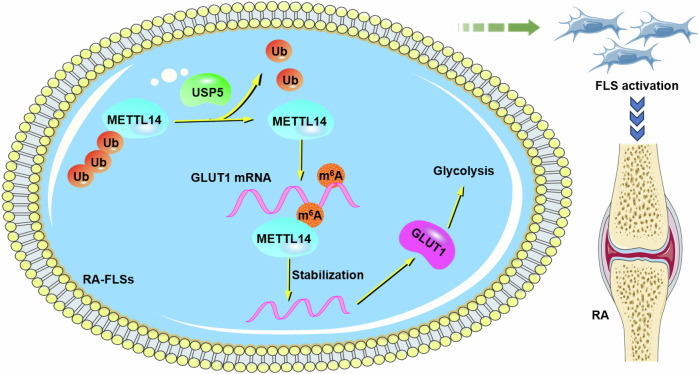
Diagram of the proposed role of USP5 in regulating glycolytic metabolism and biological behaviors of RA-FLSs. USP5 stabilizes METTL14 by inhibiting its ubiquitination, enhancing METTL14’s function in mediating m^6^A modification of GLUT1 mRNA. This modification increases the expression of GLUT1, promoting glucose uptake and glycolysis, which in turn drives the activation, proliferation, and inflammatory response of FLSs in RA. Targeting the USP5/METTL14/GLUT1 axis offers a potential therapeutic strategy for mitigating synovial inflammation in RA.

This study reveals a novel pathway wherein USP5 stabilizes METTL14 through the inhibition of METTL14 ubiquitination, allowing METTL14 to enhance m^6^A modification of GLUT1 mRNA and ultimately enhancing GLUT1-dependent glycolysis and inflammatory activation in RA-FLSs (Fig. 7). The identification of the USP5/METTL14/GLUT1 axis provides new insights into the metabolic reprogramming in RA and suggests a potential therapeutic target. These findings underscore the clinical significance of targeting metabolic pathways, as interfering with glycolytic pathways may offer a promising strategy to alleviate inflammation and prevent joint destruction in RA patients. Our results lay the groundwork for future studies focusing on metabolic interventions, offering potential advancements in RA treatment that could be integrated with current anti-inflammatory therapies.

## Materials and methods

### Experimental animals

Twenty-four male Sprague–Dawley rats (6–8 weeks old, 200–220 × *g*) were obtained from Beijing Vital River Laboratory Animal Technology Co., Ltd. (Beijing, China). Animals were housed under specific pathogen-free conditions with a 12-h light/dark cycle and free access to food and water. All procedures were approved by the Institutional Animal Care and Use Committee of Hunan Cancer Hospital, and animal studies were conducted in accordance with the ARRIVE guidelines.

### Human synovial tissue samples

Synovial tissue specimens were collected from patients with RA (*n* = 3) undergoing joint replacement surgery or knee synovectomy at our hospital, and from non-RA patients with traumatic joint injury (*n* = 3) undergoing arthroscopic surgery, serving as controls. RA patients fulfilled the 2010 American College of Rheumatology criteria for RA [41], and controls had no other joint abnormalities or systemic diseases. The study protocol was approved by the Institutional Ethics Committee, and written informed consent was obtained from all participants in accordance with the Declaration of Helsinki principles (2013).

### Animal model and treatment

The RA model was established using collagen-induced arthritis, following previously described protocols [42]. Specifically, bovine type II collagen (Chondrex, USA) was emulsified in Freund’s incomplete adjuvant (Sigma-Aldrich, USA). The mixture was administered intradermally at the base of the rats’ tails to induce immunization. Seven days later, a booster injection of type II collagen emulsified in Freund’s incomplete adjuvant was given. Then, adenovirus vectors carrying shRNA targeting USP5 (sh-USP5: 5′-GGAGATCCGGGAACGGCAGAA-3′) or its negative control sh-NC (Hanbio, Shanghai, China) were injected into the ankle joint synovium at a dose of 1 × 10^8^ plaque-forming units. The control group received equivalent injections of saline. On day 35 following the initial collagen immunization, all rats in the experimental groups were sacrificed. In this study, animals were randomly allocated into experimental groups.

### Paw thickness and arthritis scoring

Paw thickness was measured using a digital caliper every five days throughout the experiment to monitor joint swelling [43]. Clinical arthritis scores [44] were determined using a 0–4 scale for each paw (0 = no redness and swelling, 1 = mild swelling or red spot, 2 = mild swelling and red skin, 3 = erythema and moderate swelling, and 4 = erythema and severe swelling). A maximum score of 16 per animal was obtained by summing the scores of all four limbs.

### H&E staining

Synovial tissues were harvested, fixed in 4% paraformaldehyde, and dehydrated through an alcohol gradient for histopathological analysis. After dehydration, the tissues were embedded in paraffin. Thin sections, 5 µm in thickness, were prepared and stained with hematoxylin and eosin (H&E; Sigma-Aldrich, USA). A light microscope was used to capture images to evaluate synovial hyperplasia and inflammatory cell infiltration.

### Immunofluorescence

Synovial tissue sections embedded in paraffin were first treated with xylene for deparaffinization and then rehydrated through a graded ethanol series. Antigen retrieval was subsequently carried out in citrate buffer (pH 6.0) using a microwave. To block nonspecific binding, the sections were treated with 5% bovine serum albumin for 1 h. Subsequently, they were incubated overnight at 4 °C with primary antibodies, including GLUT1 (1 µg/mL, ab115730, Abcam, USA), USP5 (1:50, ab154170, Abcam), and Vimentin (2 µg/mL, ab92547, Abcam). Following washing, the sections were exposed to Alexa Fluor-conjugated secondary antibodies (Abcam) for 1 h. DAPI (Sigma-Aldrich) was used to stain the nuclei, and fluorescence microscopy was employed to capture the images. All antibodies were commercially obtained and validated by the suppliers for the immunofluorescence application.

### Cell culture and treatment

Human RA-FLSs obtained from Pricella Biotech Co., Ltd. (CP-H248, Wuhan, China), which performed mycoplasma contamination test, Vimentin immunofluorescence authentication, and STR authentication, were cultured in a specialized medium (CM-H248, Pricella) at 37 °C with 5% CO_2_. For the treatment with TNF-α or 2-DG, RA-FLSs were exposed to TNF-α (10 ng/mL; Sigma-Aldrich) or 2-DG (5 mM; Sigma-Aldrich) for 24 h to induce an inflammatory response or to inhibit glycolysis.

### Cell transfection

The shRNA targeting USP5 (sh-USP5: 5′-GATAGACATGAACCAGCGGAT-3′) or METTL14 (sh-METTL14: 5′-CCAGTTACTTACAAGCCGATA-3′), along with a negative control shRNA (sh-NC), were obtained from Hanbio (Shanghai, China). The cDNA of METTL14 or GLUT1 was cloned into the pcDNA3.1 expression vector (RiboBio, Guangzhou, China) to achieve overexpression, with the empty vector serving as the control (oe-NC). Lipofectamine 3000 (Invitrogen, Carlsbad, CA, USA) was utilized to transfect RA-FLSs with shRNA (100 nM) or pcDNA3.1 (30 nM), along with their corresponding negative controls. Transfection efficiency was confirmed 48 h post-transfection using qPCR and Western blotting.

### qPCR assay

Total RNA was extracted from RA-FLSs using Trizol reagent (Invitrogen). RNA (1 µg) was reverse transcribed to cDNA using a Reverse Transcription Kit (TaKaRa Bio, Japan). qPCR was performed with SYBR Green Master Mix (TaKaRa Bio) on an ABI 7500 Fast Real-Time PCR System (Applied Biosystems, USA). The expression levels of GLUT1 mRNA were normalized to β-actin, and relative expression was calculated using the 2^−ΔΔCt^ method. The primer sequences used for qPCR were as follows: GLUT1 forward 5′-TTG CAG GCT TCT CCA ACT GGA C-3′, and reverse 5′-CAG AAC CAG GAG CAG CTG AAG-3′; β-actin forward 5′-CCC TGG AGA AGA GCT ACG AG-3′, and reverse 5′-CGT ACA GGT CTT TGC GGA TG-3′.

### Western blot

Proteins from RA-FLSs and synovial tissues were extracted using RIPA buffer (Thermo Fisher Scientific, USA), and their concentrations were quantified using the BCA protein assay kit (Beyotime, Shanghai, China). Proteins (30 µg per sample) were equally loaded, separated by SDS-PAGE, and transferred onto PVDF membranes (Beyotime). The membranes were incubated in 5% non-fat dry milk prepared in TBS-T for 1 h to block nonspecific binding, followed by overnight incubation at 4 °C with primary antibodies primary antibodies against METTL14 (1:500, ab308576, Abcam), USP5 (1:1000, ab154170, Abcam), GLUT1 (1:100000, ab115730, Abcam), and β-actin (1:1000, ab8227, Abcam, USA). All antibodies were commercially obtained and validated by the suppliers for the Western blot application. The membranes were washed and then incubated with HRP-conjugated secondary antibodies (1:10000, ab6721, Abcam) for one hour. Bands were visualized using an Enhanced Chemiluminescence (ECL) Detection Kit (Thermo Fisher Scientific). Full and uncropped western blots, uploaded as “Supplementary materials-Western blot original images”.

### CCK-8 assay

The Cell Counting Kit-8 (Solarbio, Beijing, China) was used to evaluate cell viability. RA-FLSs were seeded in 96-well plates at a density of 5 × 10^3^ cells per well and treated as indicated. After treatment, each well received 10 µL of CCK-8 solution and was incubated for 2 h at 37 °C. The absorbance at 450 nm was then measured using a BioTek microplate reader (USA).

### Transwell assay

Transwell chambers (24-well, Cornin, Kennebunk, USA) were used to evaluate cell migration and invasion. For migration assay, RA-FLSs (5 × 10^4^) were plated in the upper chamber with serum-free medium, and the lower chamber was filled with complete medium. For invasion assays, matrigel (Corning) was applied to pre-coat the transwell inserts. After 24 h of incubation at 37 °C, cells that had traversed to the underside of the membrane were fixed in methanol, stained with crystal violet, and analyzed under a microscope.

### Apoptosis detection

Apoptosis in RA-FLSs was assessed using the Annexin V-FITC/PI Apoptosis Detection Kit (BioLegend, California, USA). Cells were collected, rinsed with cold phosphate-buffered saline to eliminate residual media and impurities, and then suspended in binding buffer for further staining and examination. To this suspension, 5 μL of Annexin V-FITC and 5 μL of propidium iodide (PI) solution were added to stain the cells for apoptosis analysis. The cells were then incubated in the dark for 15 min to allow proper binding of the dyes. The samples were evaluated with a flow cytometer (BD FACSCanto II, USA), and the data were analyzed using FlowJo software.

### ELISA

The levels of inflammatory cytokines (IL-6: E-EL-H6156, IL-1β: E-EL-H0149, TNF-α: E-EL-H0109) were quantified using ELISA kits (Elabscience, Wuhan, China). Specifically, 100 μL of the sample was dispensed into pre-coated plates and incubated for 1.5 h at 37 °C. After discarding the liquid, 100 µL of biotinylated antibody working solution was added for a one-hour incubation. Following washing, 100 µL of HRP-conjugated working solution was added, followed by a 30-min incubation. Subsequently, 90 µL of substrate reagent was introduced, and the mixture was incubated for 15 min. Afterward, 50 µL of stop solution was added, and absorbance was measured at 450 nm using a microplate reader (Bio-Rad).

### Seahorse analysis

The extracellular acidification rate (ECAR), which reflects glycolytic activity, was assessed using the Seahorse XF Glycolysis Stress Test Kit (Agilent Technologies, USA) on a Seahorse XF-96 Analyzer (Seahorse Bioscience). RA-FLSs (4 × 10^4^) were seeded in Seahorse XF cell culture microplates and treated as indicated. ECAR was recorded following the sequential addition of glucose (10 mM), oligomycin (1 μM), and 2-DG (50 mM). Additionally, cell glucose uptake and lactate production were measured using Glucose Assay Kit (E1011, Applygen, Beijing, China) and Lactate Assay Kit (E-BC-K044-M, Elabscience), all in strict accordance with the manufacturers’ instructions.

### Co-immunoprecipitation (Co-IP)

Co-IP assay was conducted to assess the interaction between USP5 and METTL14. RA-FLSs were lysed using IP lysis buffer (Thermo Fisher Scientific, USA) containing protease inhibitors. The resulting lysates were incubated overnight at 4 °C with anti-USP5 antibody (ab241311, Abcam), anti-METTL14 antibody (ab309096, Abcam), or control IgG (ab172730, Abcam). All antibodies were commercially obtained and validated by the suppliers for the IP application. Following this, protein A/G magnetic beads (Thermo Fisher Scientific) were added and incubated for 2 h at 4 °C. After extensive washing, the bound proteins were eluted and analyzed by Western blot.

### Ubiquitination assay

Cells were lysed, and METTL14 was immunoprecipitated using an anti-METTL14 antibody, followed by incubation with Protein A/G beads. After washing the beads, the complexes were eluted using SDS sample buffer. The eluted proteins were subjected to SDS-PAGE, transferred onto a PVDF membrane, and blocked. A Western blot was performed using an anti-ubiquitin antibody (1:1000, ab134953, Abcam) to detect the ubiquitination status of METTL14, followed by incubation with an HRP-conjugated secondary antibody and visualization using ECL reagent. All antibodies were commercially obtained and validated by the suppliers for the indicated applications.

### Methylated RNA immunoprecipitation (MeRIP)

MeRIP assay was performed to assess the m^6^A modification of GLUT1 mRNA. Total RNA was extracted from RA-FLSs using Trizol reagent, followed by mRNA purification through poly (A) selection. mRNA (5 µg) was incubated with an m^6^A-specific antibody (202003, Synaptic Systems, Gottingen, Germany) or IgG overnight at 4 °C. Antibodies were commercially obtained and validated by the suppliers for the MeRIP application. The RNA-antibody complex was captured using protein A/G magnetic beads, and the bound RNA was eluted, purified, and subjected to qPCR analysis to determine the enrichment of m^6^A-modified GLUT1 mRNA.

### mRNA stability assay

The stability of GLUT1 mRNA was assessed using actinomycin D (Sigma-Aldrich), a transcription inhibitor. RA-FLSs were exposed to actinomycin D (5 µg/mL) for different durations (0, 4, 8 h), and total RNA was extracted at each time point. The remaining levels of GLUT1 mRNA were determined by qPCR to evaluate the impact of METTL14 on the stability of GLUT1 mRNA. The actinomycin D concentration and time points were applied with reference to a previous study [18].

### Dual-luciferase reporter assay

Predicted m⁶A motifs in GLUT1 mRNA (positions 1856, 1889, and 2111) were identified using the SRAMP online prediction tool. To examine their functional relevance, site-directed mutagenesis was performed by replacing the core adenosine (A) within each GGACU/AGACU/GAACU consensus motif with cytosine (C) or guanine (G). Three mutant constructs (Mut1, Mut2, and Mut3), along with the wild-type (WT) GLUT1 sequence, were cloned into the pmirGLO luciferase reporter vector (Promega, USA). Then, these recombinant plasmids and either sh-NC or sh-METTL14 were transfected into cells using Lipofectamine 3000 (Invitrogen). After 48 h, luciferase activity was measured with the Dual-Luciferase Reporter Assay System (Promega) according to the manufacturer’s protocol. Firefly luciferase activity was normalized to Renilla luciferase activity, and results were expressed as relative luciferase activity.

### Cycloheximide (CHX) chase assay

To assess the effect of USP5 on METTL14 protein stability, RA-FLSs were transfected with oe-NC or oe-USP5. Cells were treated with CHX (100 μg/mL; Sigma-Aldrich) for the indicated time periods (0, 4, 8, 12, 16, and 24 h), and total protein was extracted at each time point. METTL14 protein levels were analyzed by Western blot, and band intensities were quantified by densitometry. Protein stability was evaluated by plotting the percentage of remaining METTL14 protein relative to time 0 h.

### Statistical analysis

All data are expressed as the mean ± standard deviation (SD). The results of cell culture and human sample experiments were obtained from at least three independent replicates. For animal study, six mice per group were used. The sample size was predetermined based on published literatures and previous lab experience. No statistical methods were used to predetermine the sample size. No samples were excluded from the analysis. Statistical analysis and graphing were performed using GraphPad Prism 8.0 (GraphPad Software, Inc., San Diego, CA). Differences among multiple groups or between two groups were assessed using one-way ANOVA followed by Tukey’s *post hoc* test or Student’s *t* test. The investigator was blinded to the group allocation during the experiment and/or when assessing the outcome. Normal distribution of data was verified using a Shapiro–Wilkinson test. The variance was similar between the groups that were being statistically compared. A p-value of less than 0.05 was considered statistically significant.

## Supplementary information

Figure S1

Figure S2

Supplementary figure legends

aj-checklist

Western blot original images

## Data Availability

The datasets used or analyzed during the current study are available from the corresponding author on reasonable request.
