# Supplementary figures and images for: USP5 promotes glycolysis of fibroblast-like synoviocytes by stabilizing the METTL14/m6A/GLUT1 axis in rheumatoid arthritis
Source: Cell Death Discov. 2025 Dec 3;12:32. doi: 10.1038/s41420-025-02890-2 (PMC12811265; doi:10.1038/s41420-025-02890-2)

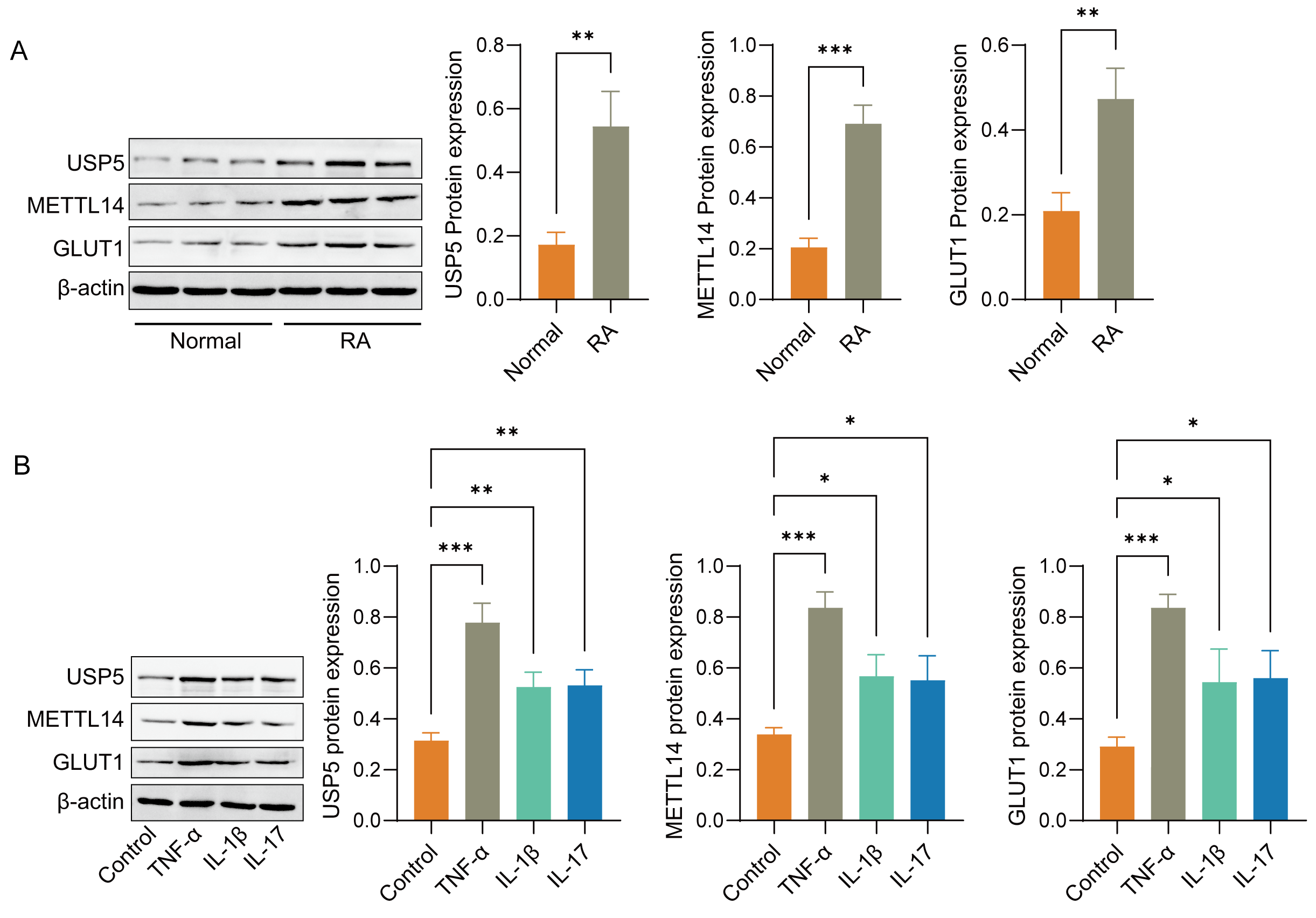

Supplement: Supplementary file 1 — Figure S1 [file 41420_2025_2890_MOESM1_ESM.tif]

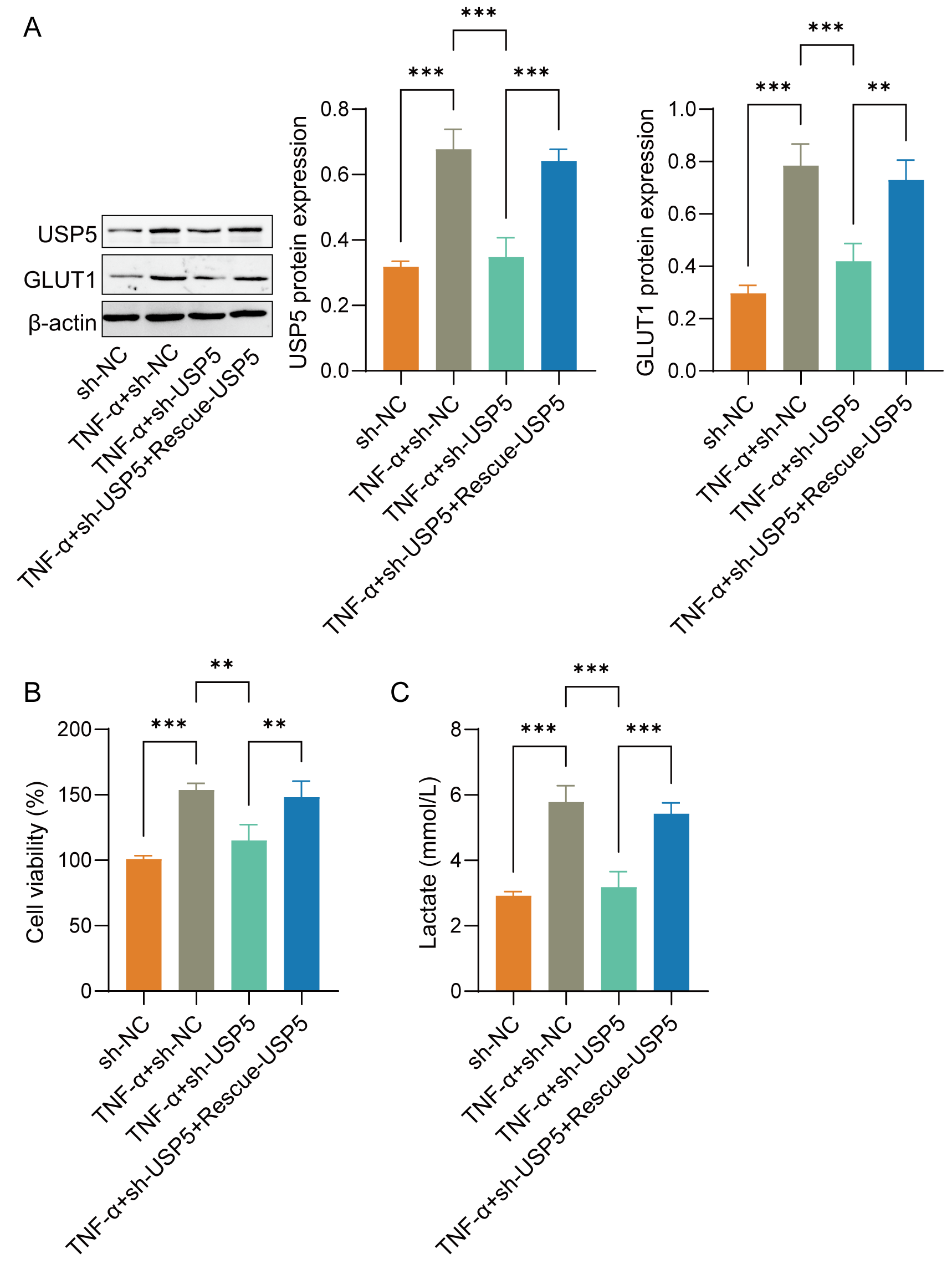

Supplement: Supplementary file 2 — Figure S2 [file 41420_2025_2890_MOESM2_ESM.tif]
