# Supplementary material for: USP5 promotes glycolysis of fibroblast-like synoviocytes by stabilizing the METTL14/m6A/GLUT1 axis in rheumatoid arthritis
Source: Cell Death Discov. 2025 Dec 3;12:32. doi: 10.1038/s41420-025-02890-2 (PMC12811265; doi:10.1038/s41420-025-02890-2)
Supplement: Supplementary file 3 — Supplementary figure legends [file 41420_2025_2890_MOESM3_ESM.docx]

**Supplementary figure legends**

**Figure S1. The expression of USP5, METTL14, and GLUT1 in RA synovial tissues and inflammatory cytokine-induced RA-FLSs.** (A) Western blot analysis of USP5, METTL14, and GLUT1 expression in human synovial tissues from RA patients and normal controls. (B) RA-FLSs were treated with TNF-α, IL-1β, or IL-17 (10 ng/mL, 24 hours), and protein expression levels of USP5, METTL14, and GLUT1 were examined by Western blot. N = 3. *p <0.05, **p <0.01, ***p <0.001.

**Figure S2. Rescue experiments confirm the specificity of sh-USP5.** RA-FLSs were divided into four groups (sh-NC, TNF-α+sh-NC, TNF-α+sh-USP5, TNF-α+sh-USP5+Rescue-USP5). Rescue-USP5, a target gene expression vector containing silent mutations. Western blot (A), CCK-8 (B), and lactate (C) assays showed that Rescue-USP5 restored USP5/GLUT1 expression, cell viability, and lactate production suppressed by sh-USP5. N = 3. **p <0.01, ***p <0.001.
