# Supplementary material for: USP5 promotes glycolysis of fibroblast-like synoviocytes by stabilizing the METTL14/m6A/GLUT1 axis in rheumatoid arthritis
Source: Cell Death Discov. 2025 Dec 3;12:32. doi: 10.1038/s41420-025-02890-2 (PMC12811265; doi:10.1038/s41420-025-02890-2)
Supplement: Supplementary file 5 — Western blot original images [file 41420_2025_2890_MOESM5_ESM.pptx]

## Slide 1
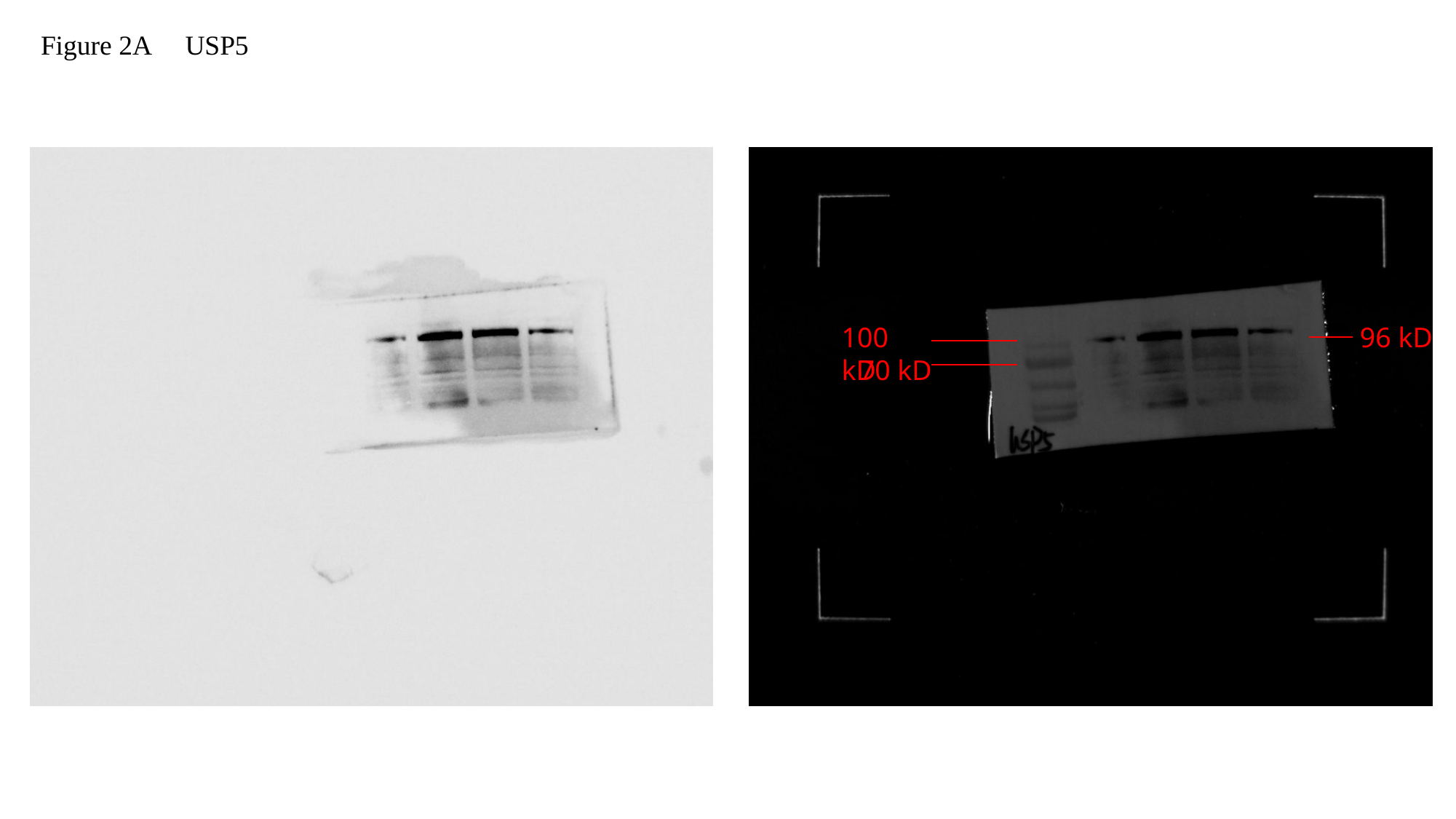

Figure 2A USP5
100 kD
96 kD
70 kD

## Slide 2
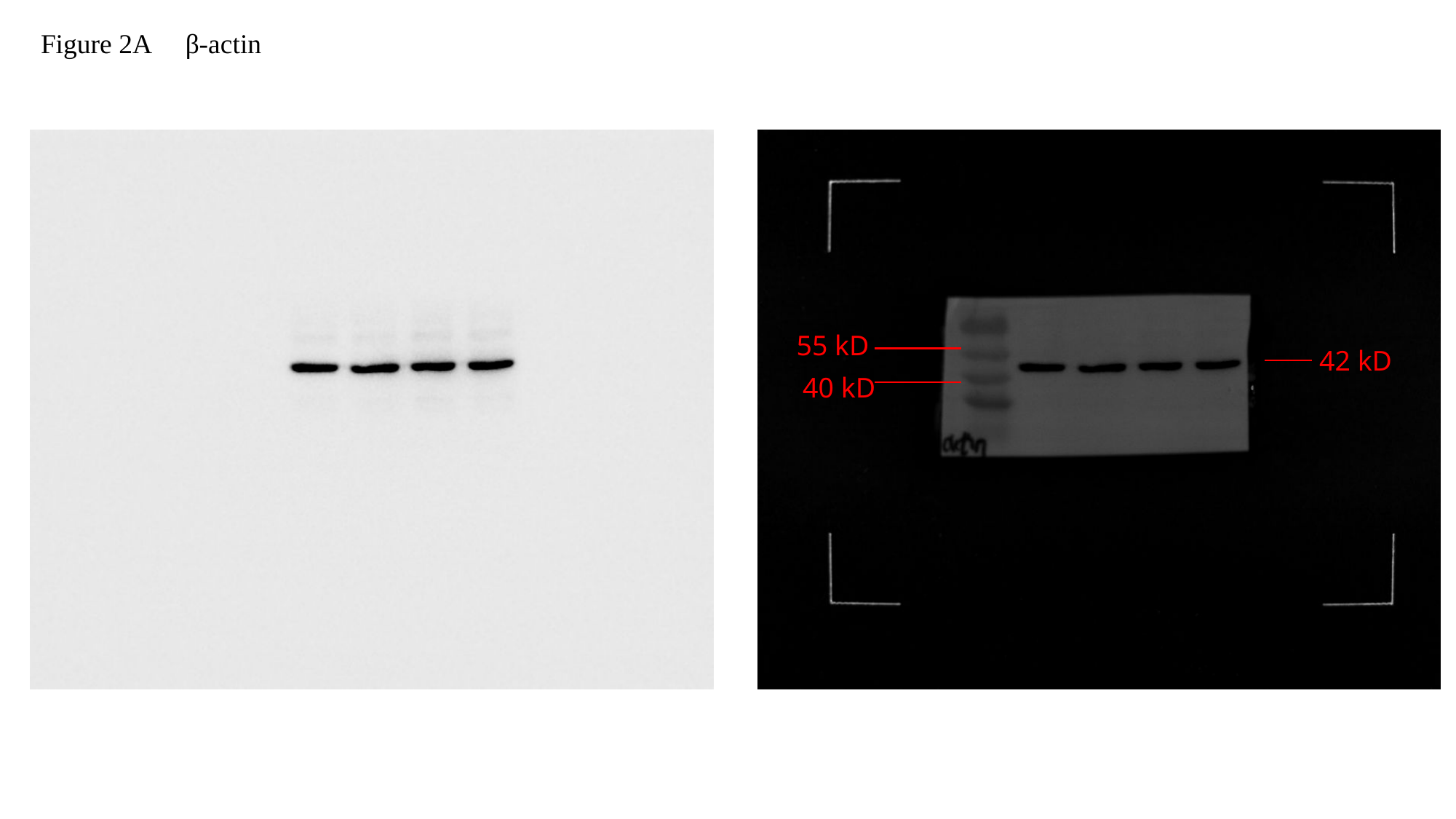

Figure 2A β-actin
55 kD
42 kD
40 kD

## Slide 3
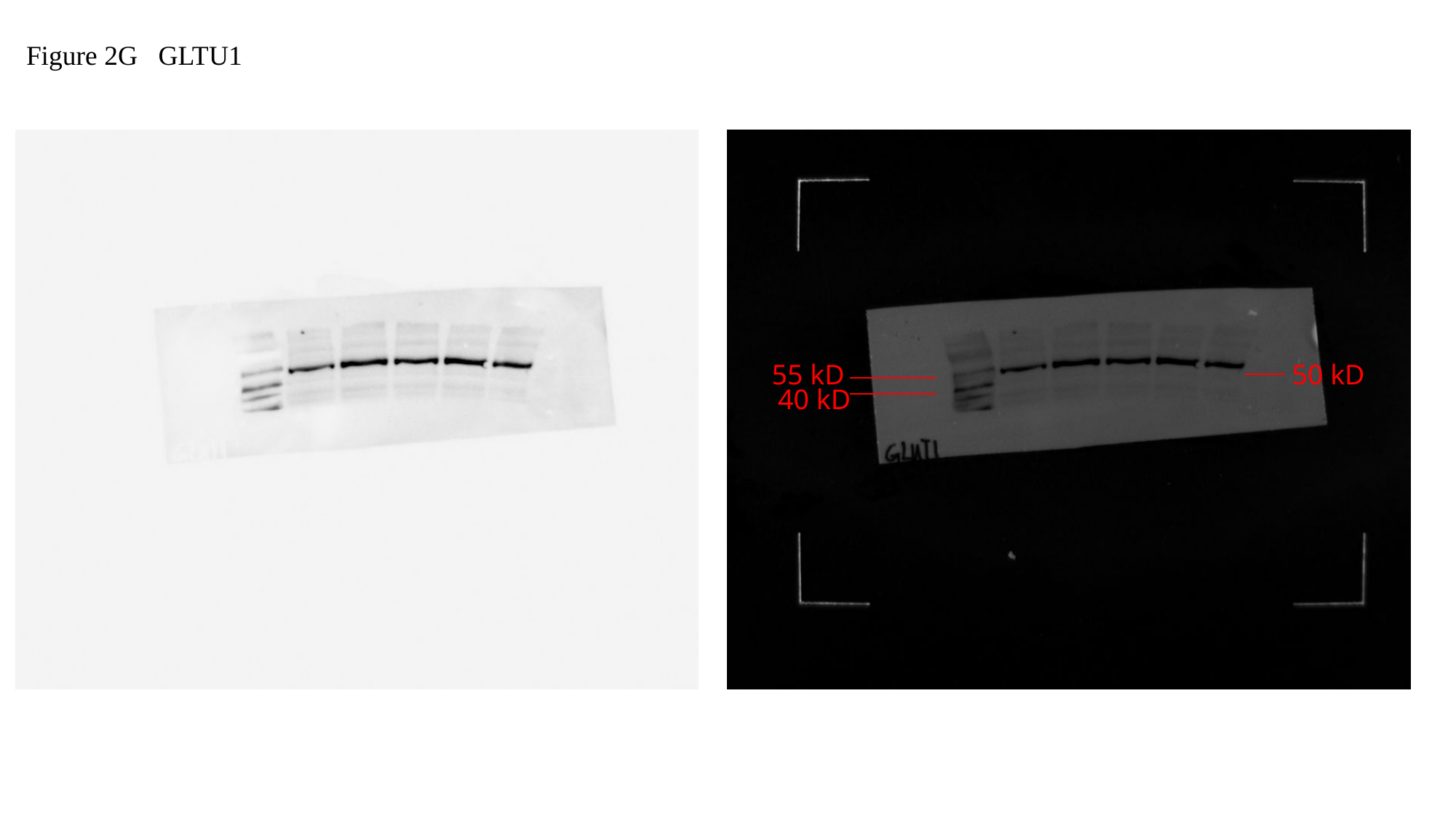

Figure 2G GLTU1
55 kD
50 kD
40 kD

## Slide 4
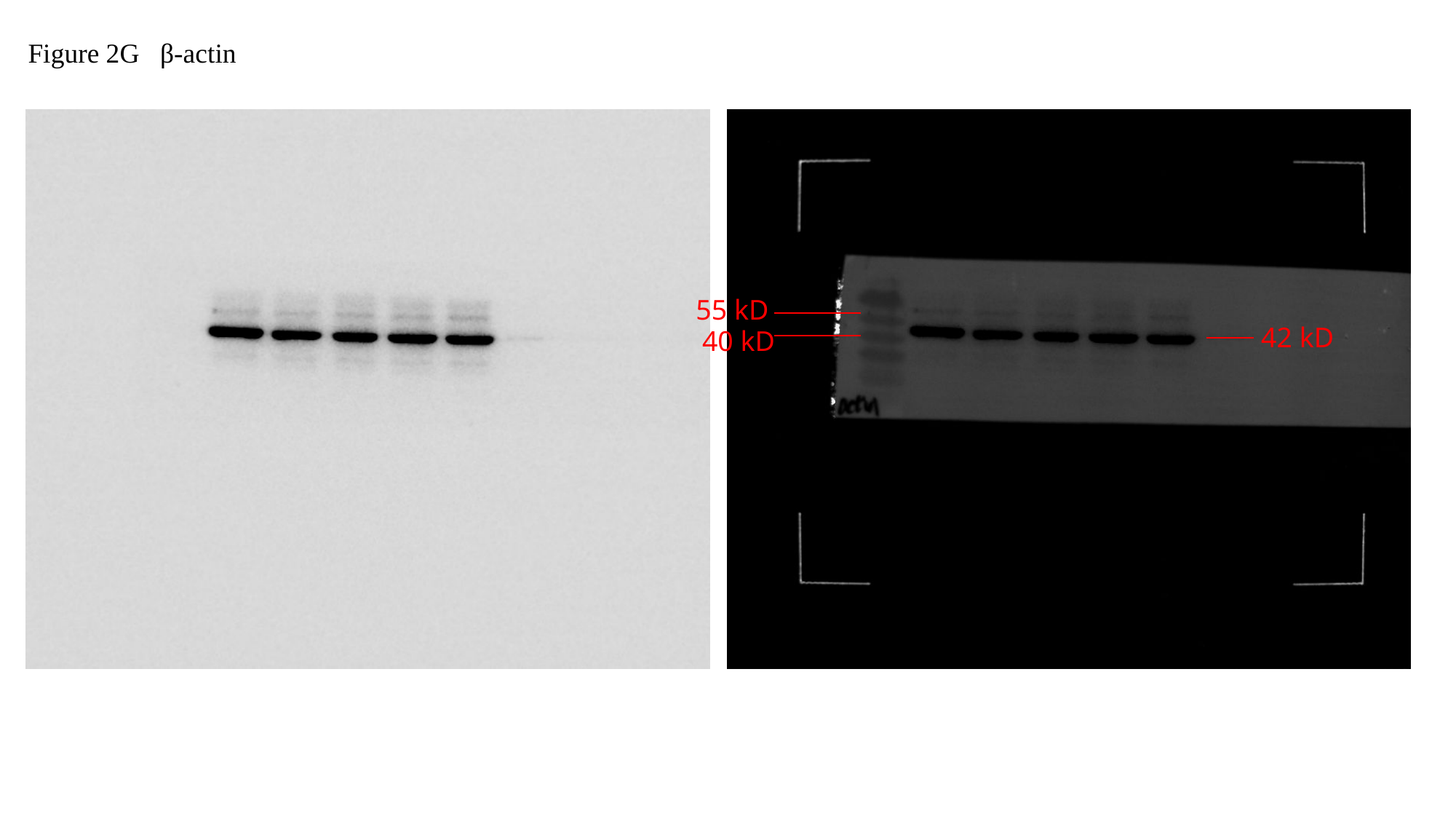

Figure 2G β-actin
55 kD
42 kD
40 kD

## Slide 5
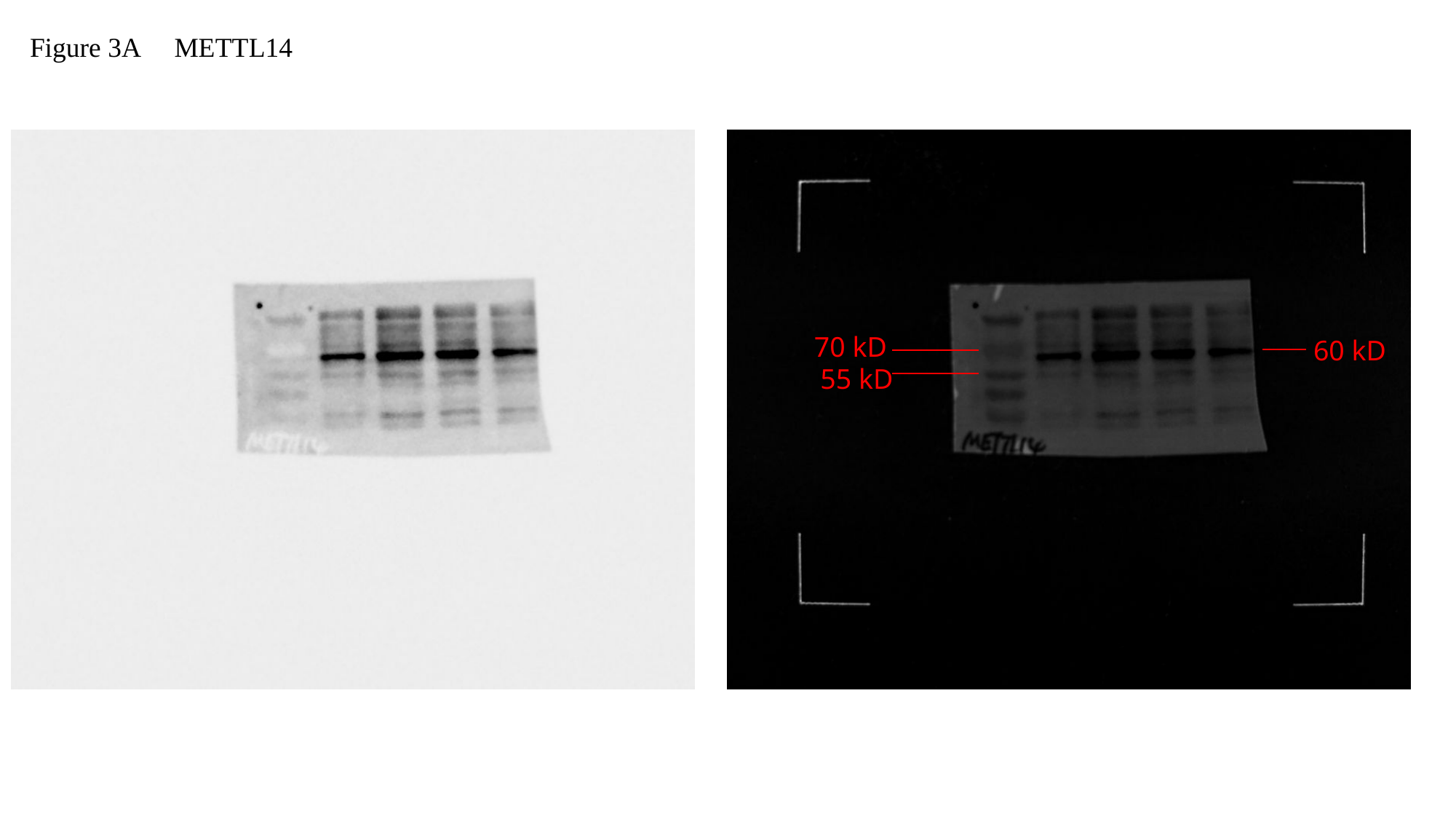

Figure 3A METTL14
70 kD
60 kD
55 kD

## Slide 6
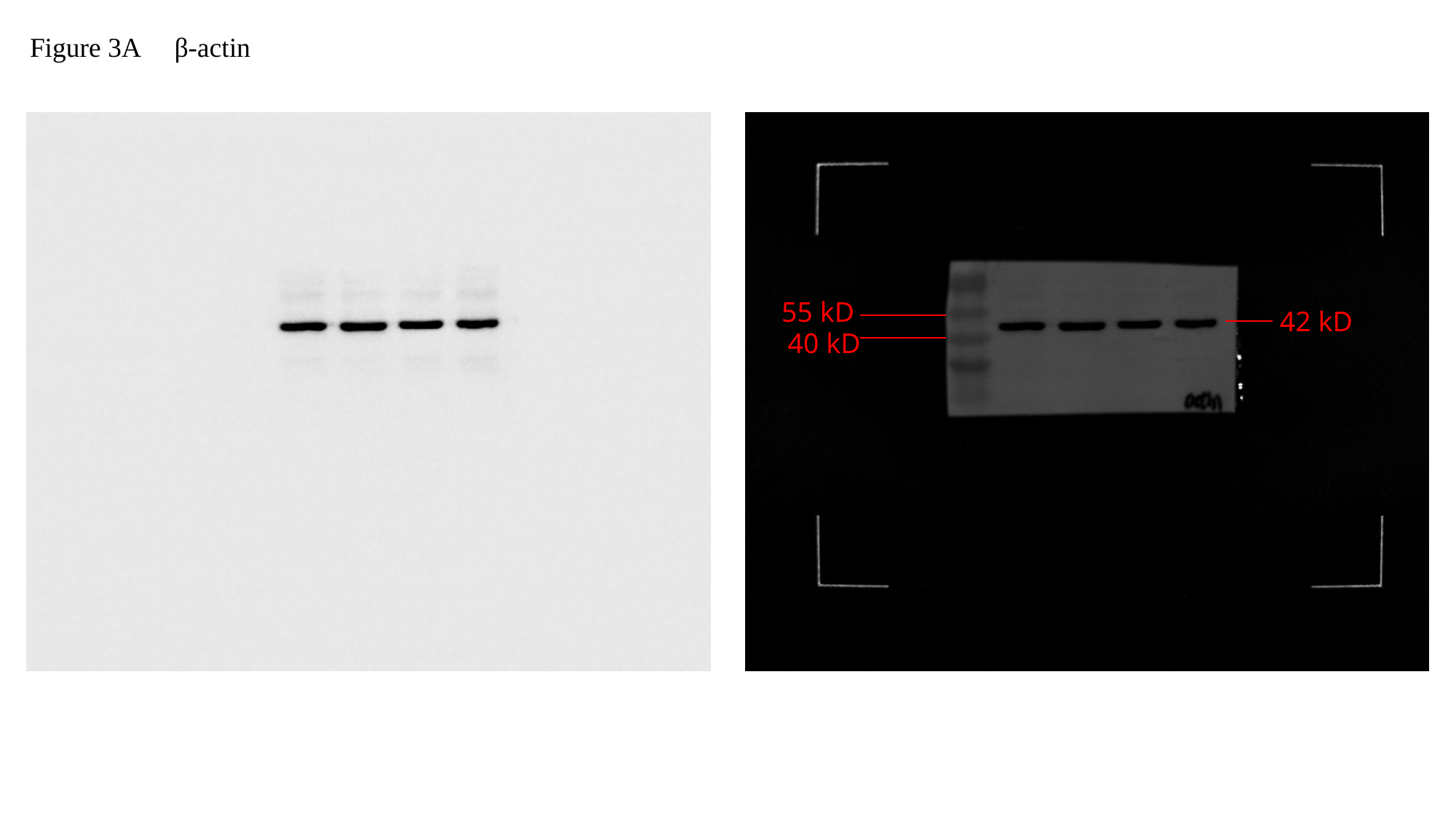

Figure 3A β-actin
55 kD
42 kD
40 kD

## Slide 7
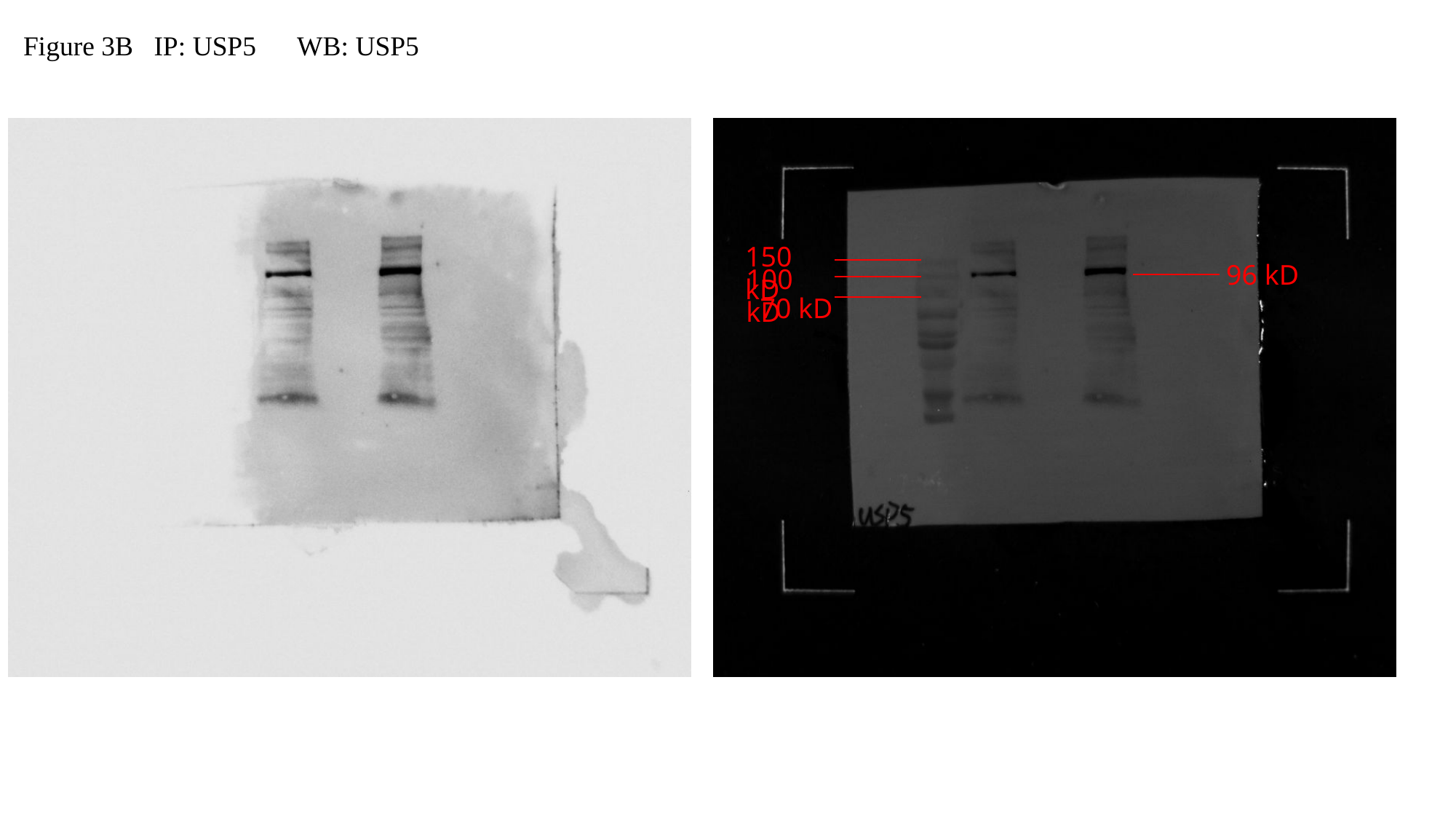

Figure 3B IP: USP5 WB: USP5
150 kD
96 kD
100 kD
70 kD

## Slide 8
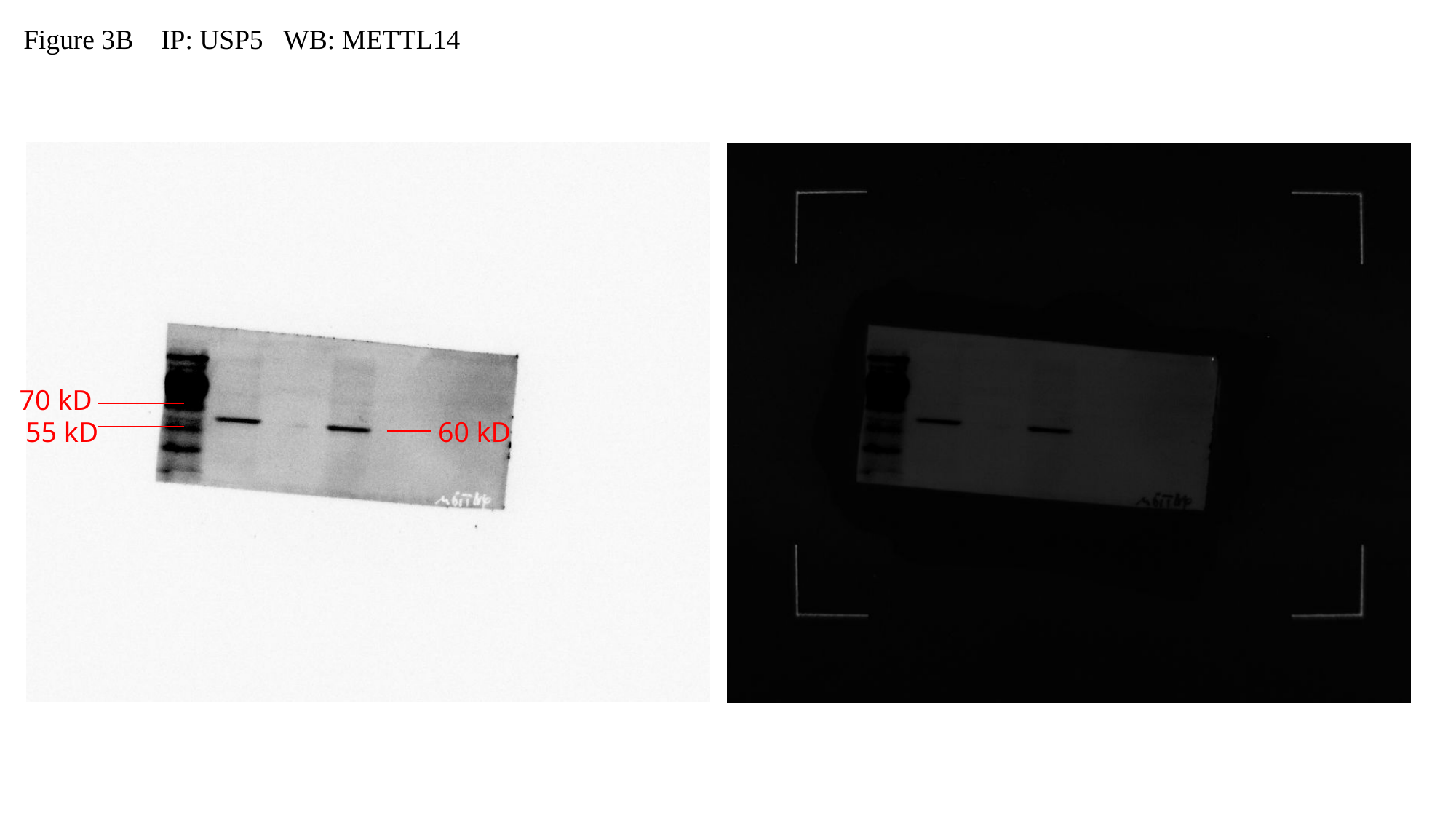

Figure 3B IP: USP5 WB: METTL14
70 kD
55 kD
60 kD

## Slide 9
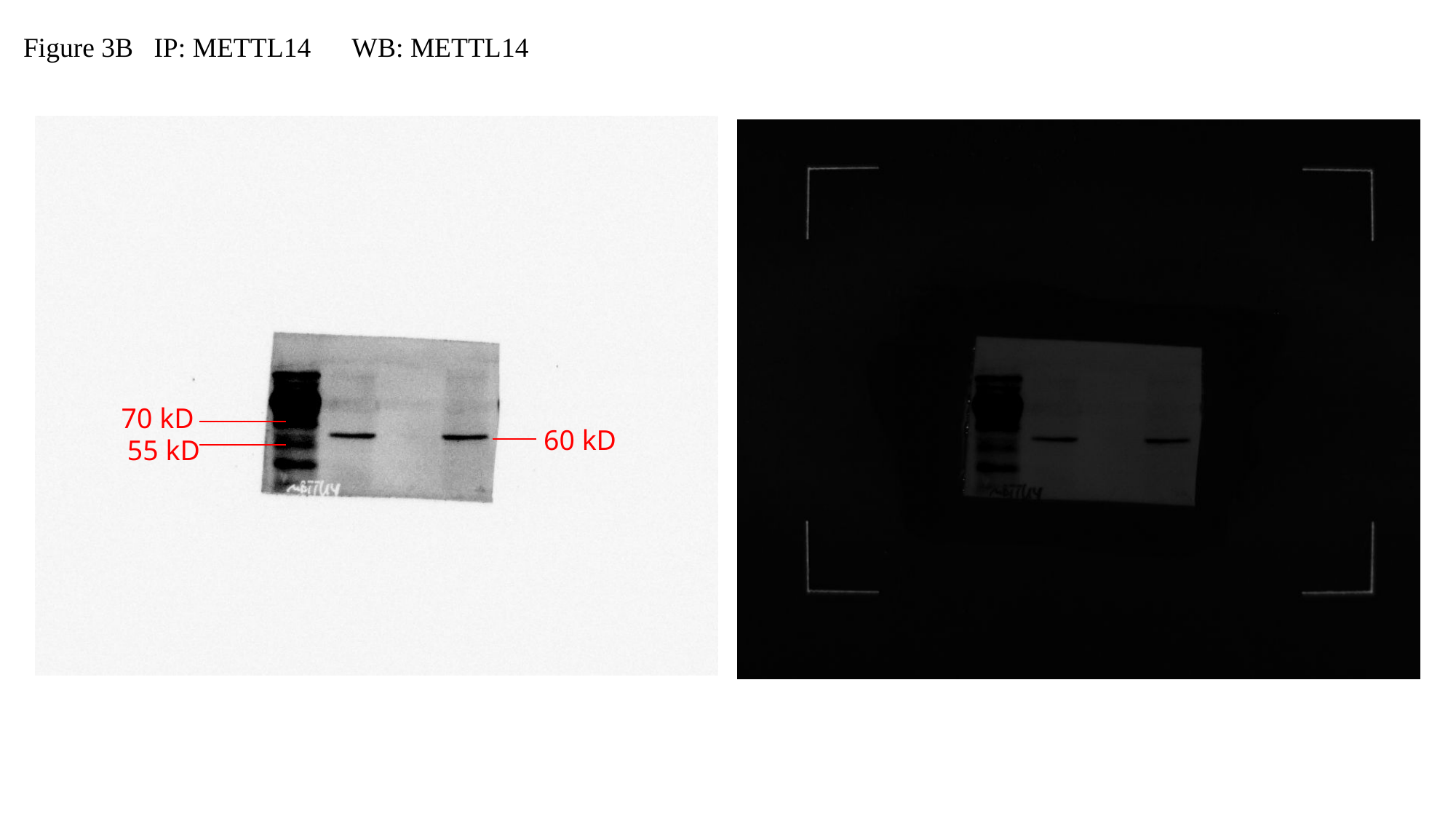

Figure 3B IP: METTL14 WB: METTL14
70 kD
60 kD
55 kD

## Slide 10
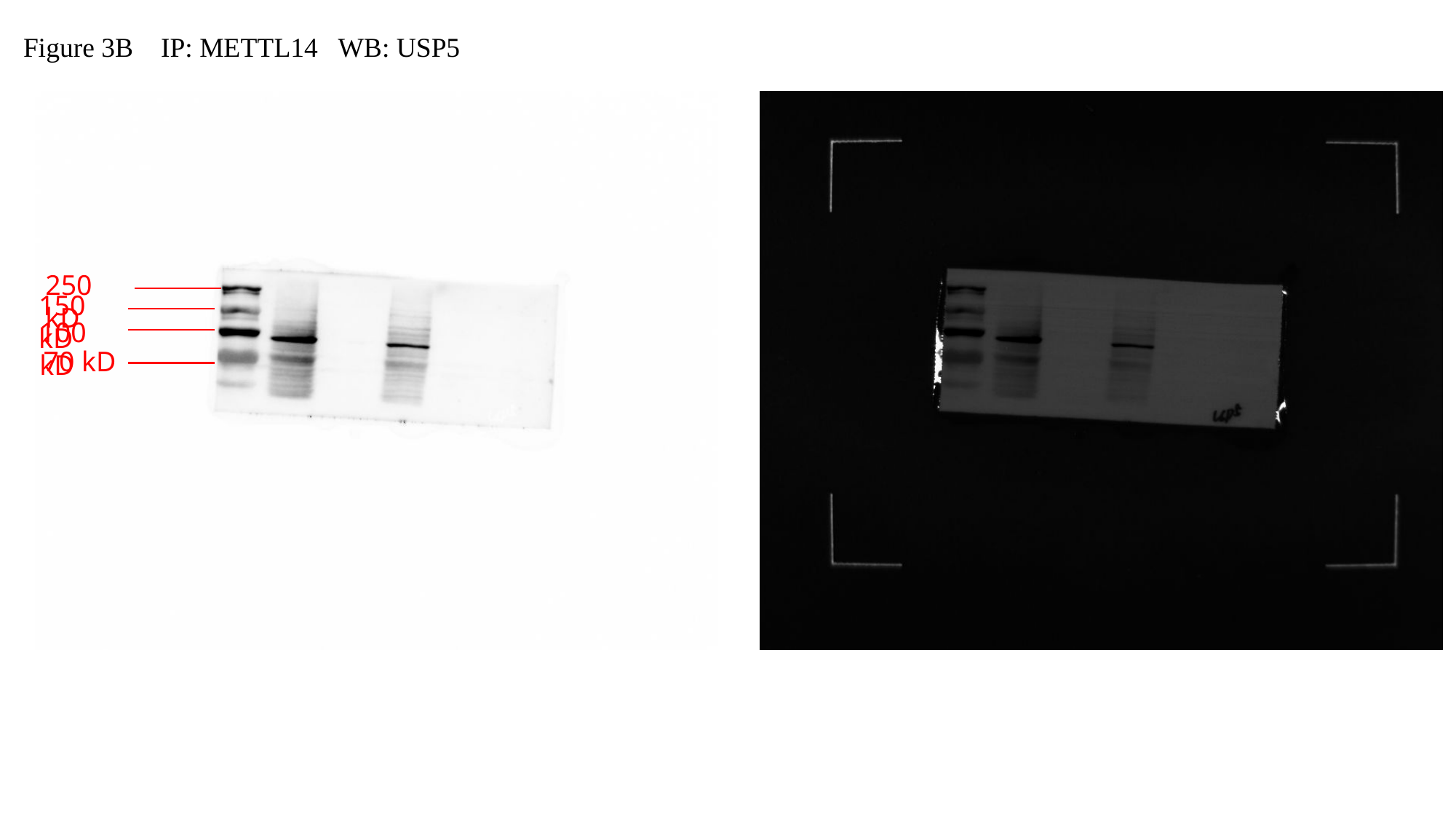

Figure 3B IP: METTL14 WB: USP5
250 kD
150 kD
100 kD
70 kD

## Slide 11
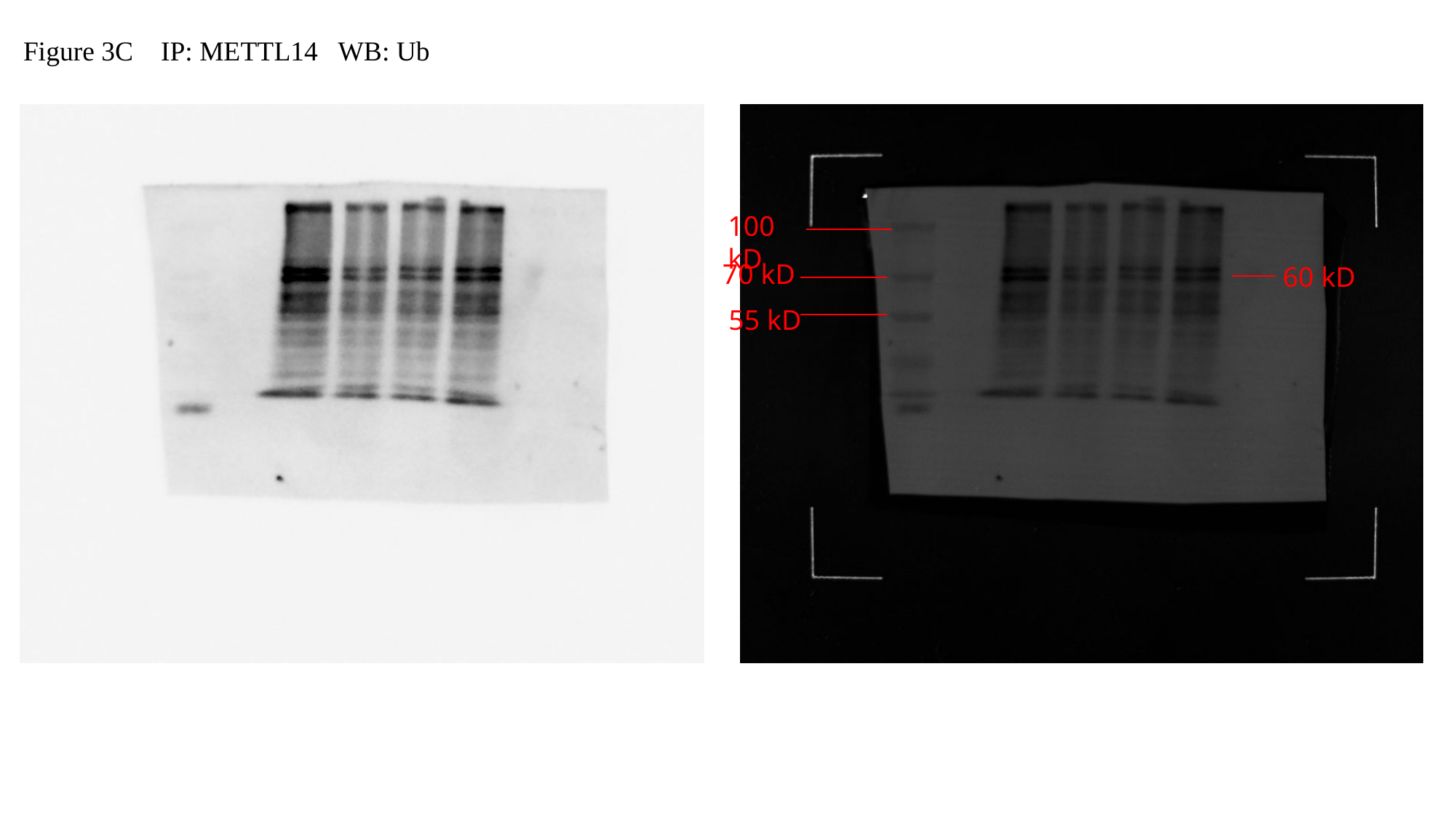

Figure 3C IP: METTL14 WB: Ub
100 kD
70 kD
60 kD
55 kD

## Slide 12
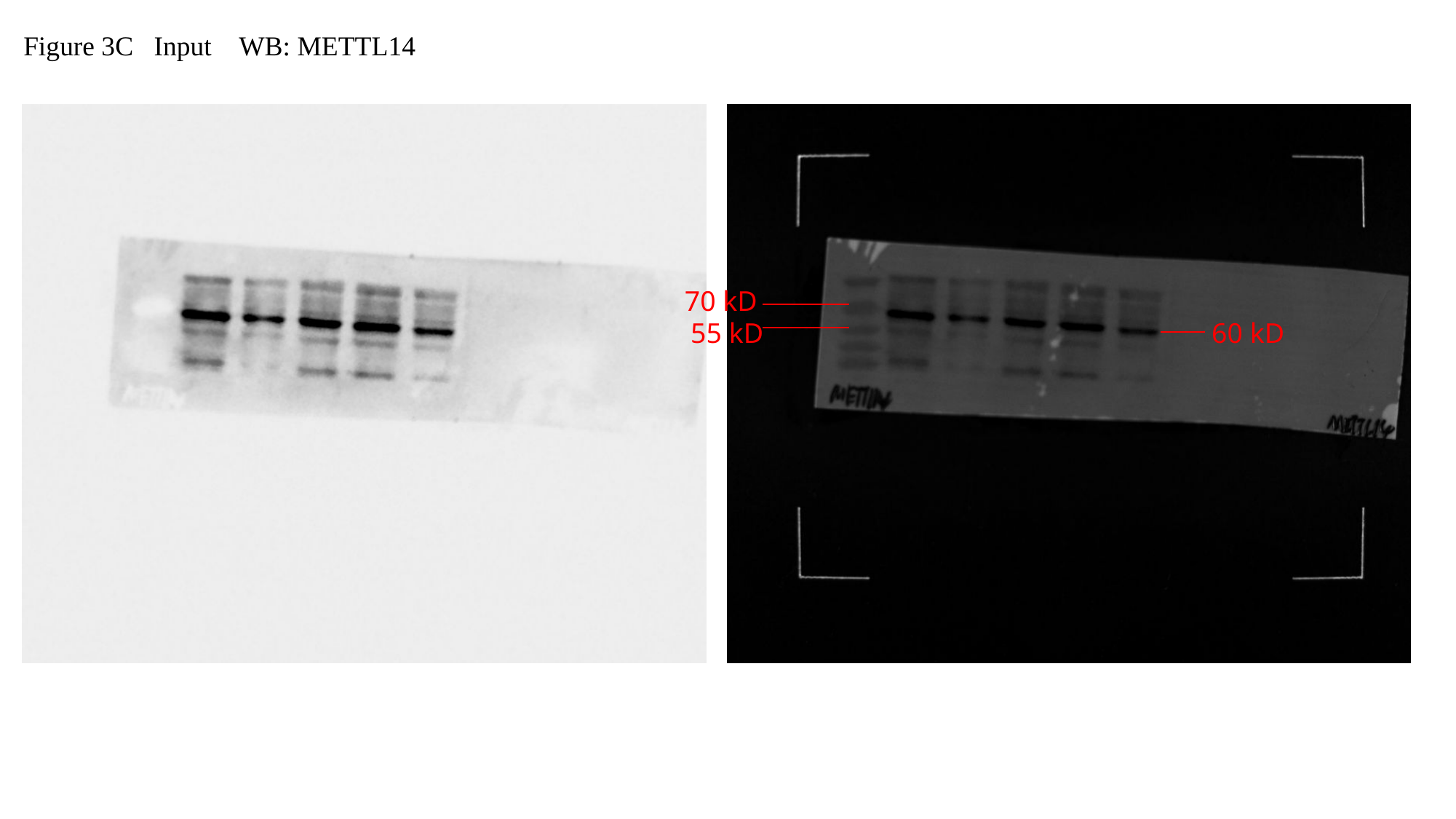

Figure 3C Input WB: METTL14
70 kD
55 kD
60 kD

## Slide 13
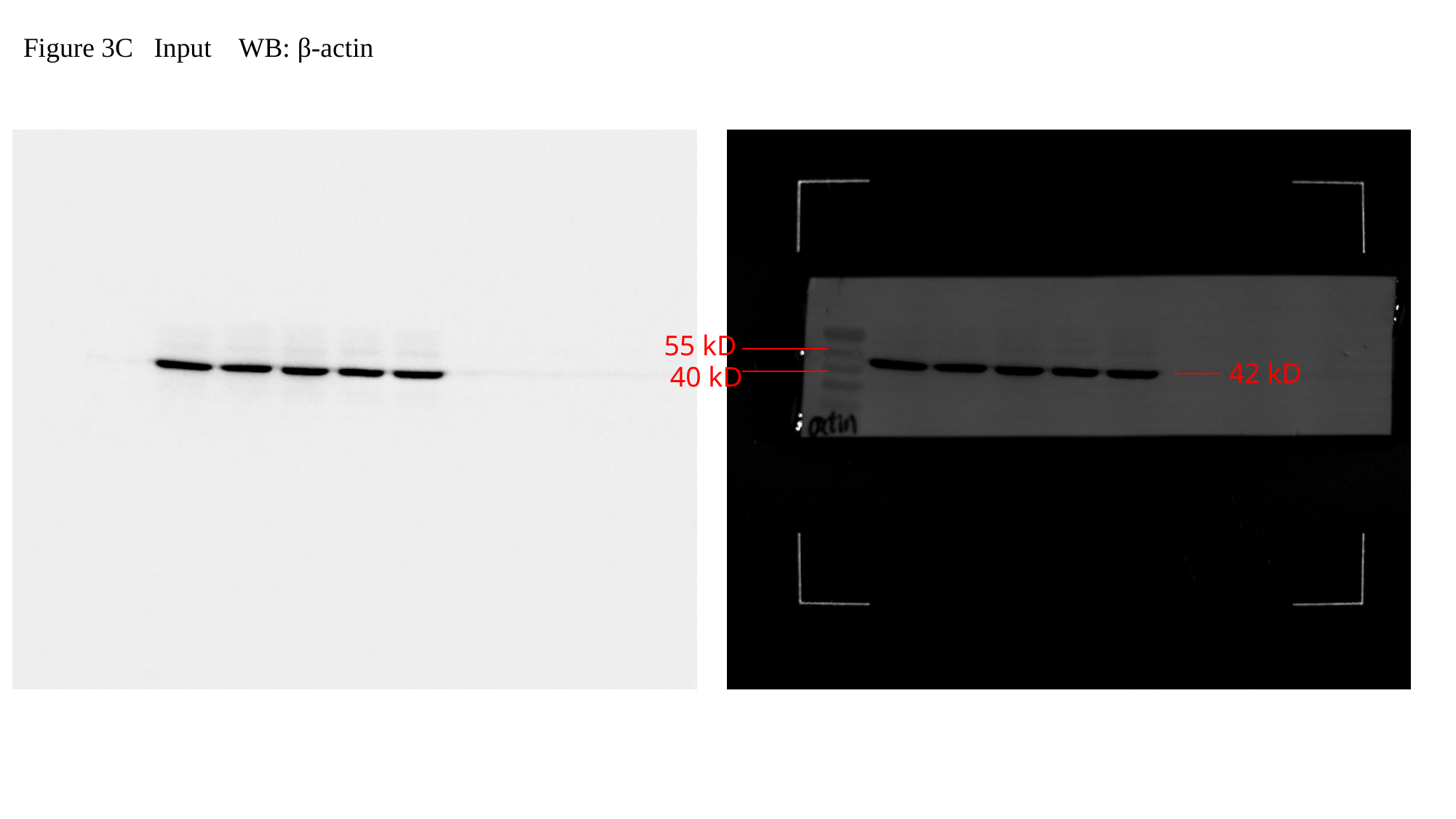

Figure 3C Input WB: β-actin
55 kD
42 kD
40 kD

## Slide 14
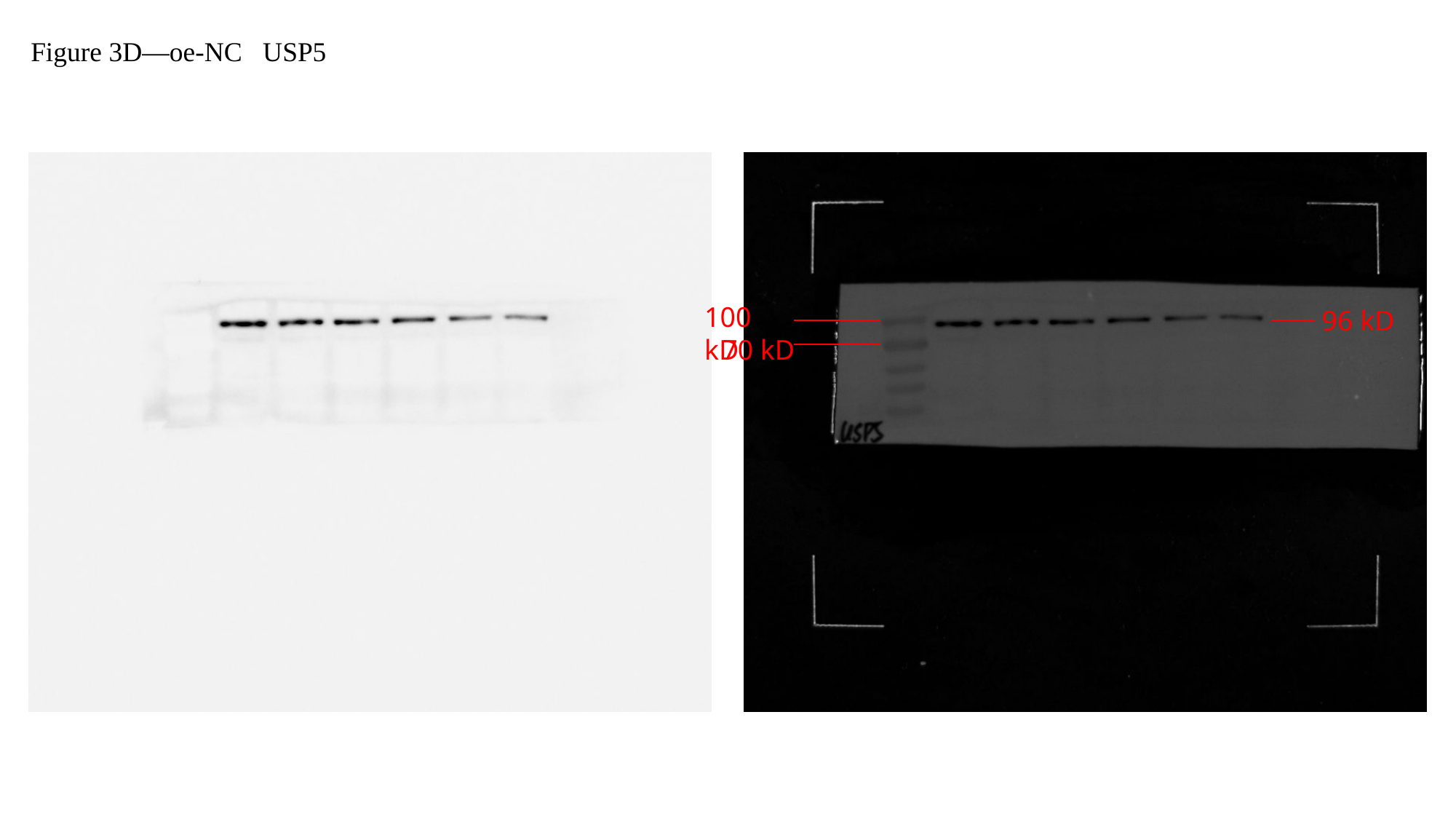

Figure 3D—oe-NC USP5
100 kD
96 kD
70 kD

## Slide 15
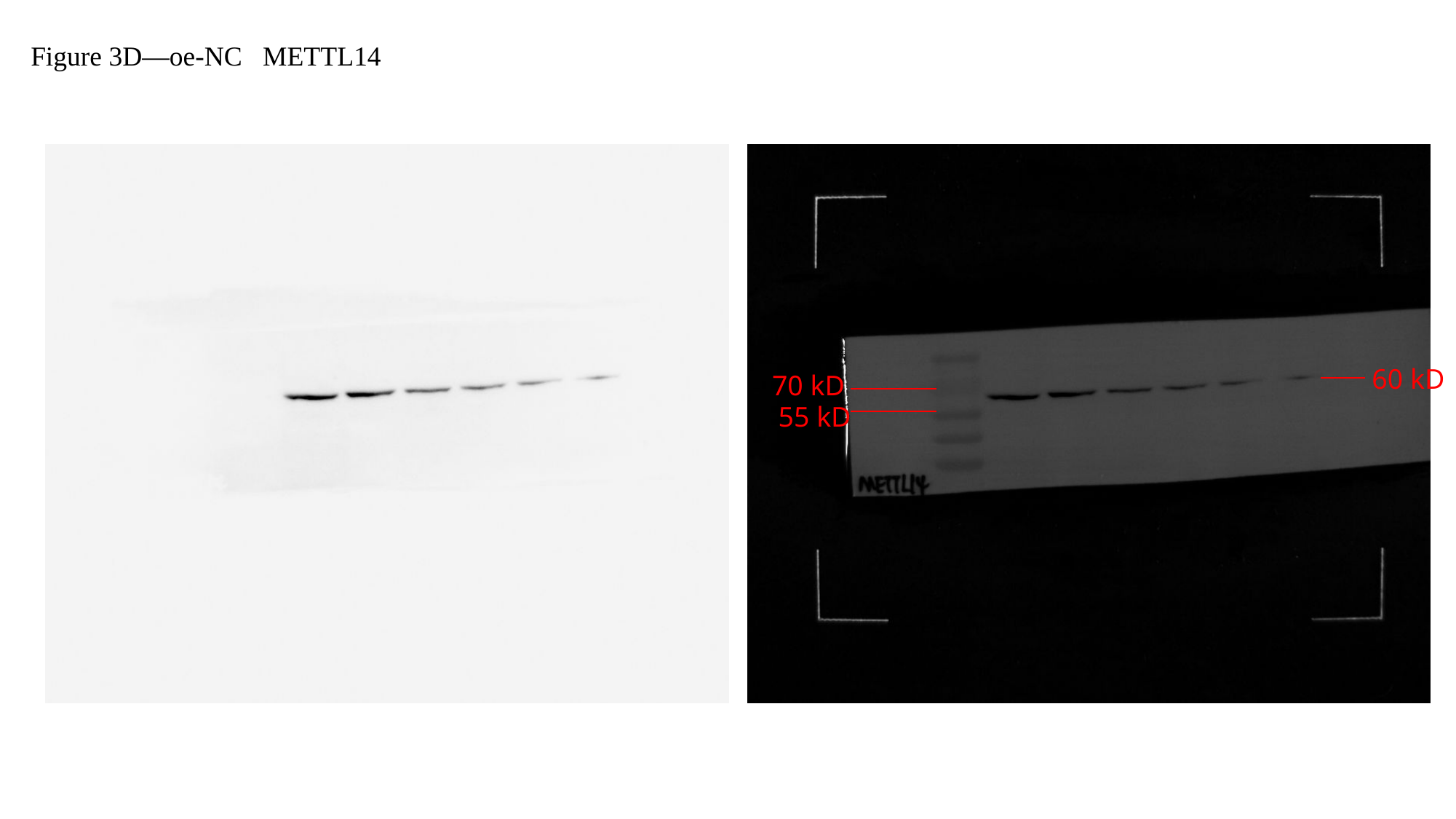

Figure 3D—oe-NC METTL14
60 kD
70 kD
55 kD

## Slide 16
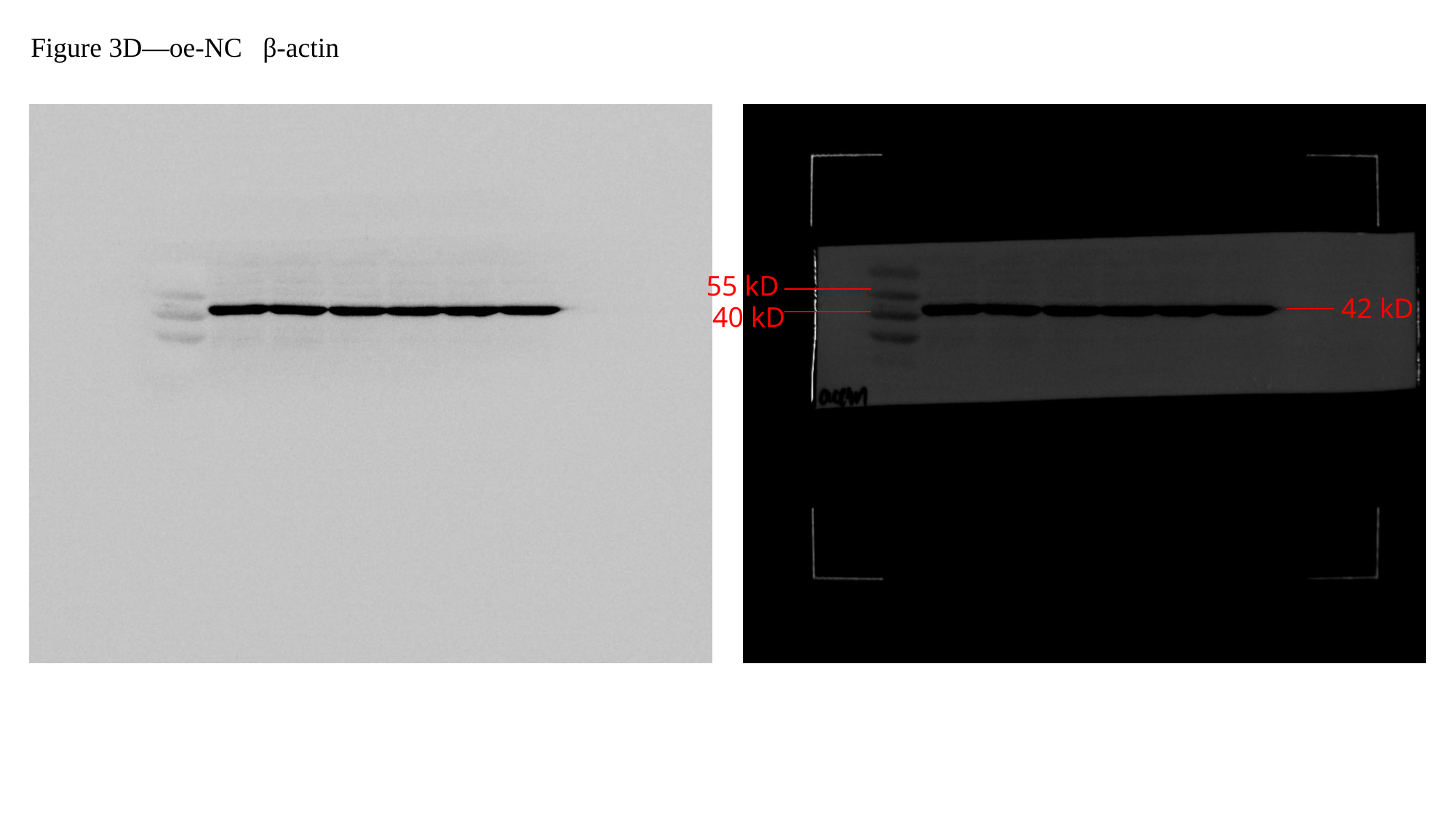

Figure 3D—oe-NC β-actin
55 kD
42 kD
40 kD

## Slide 17
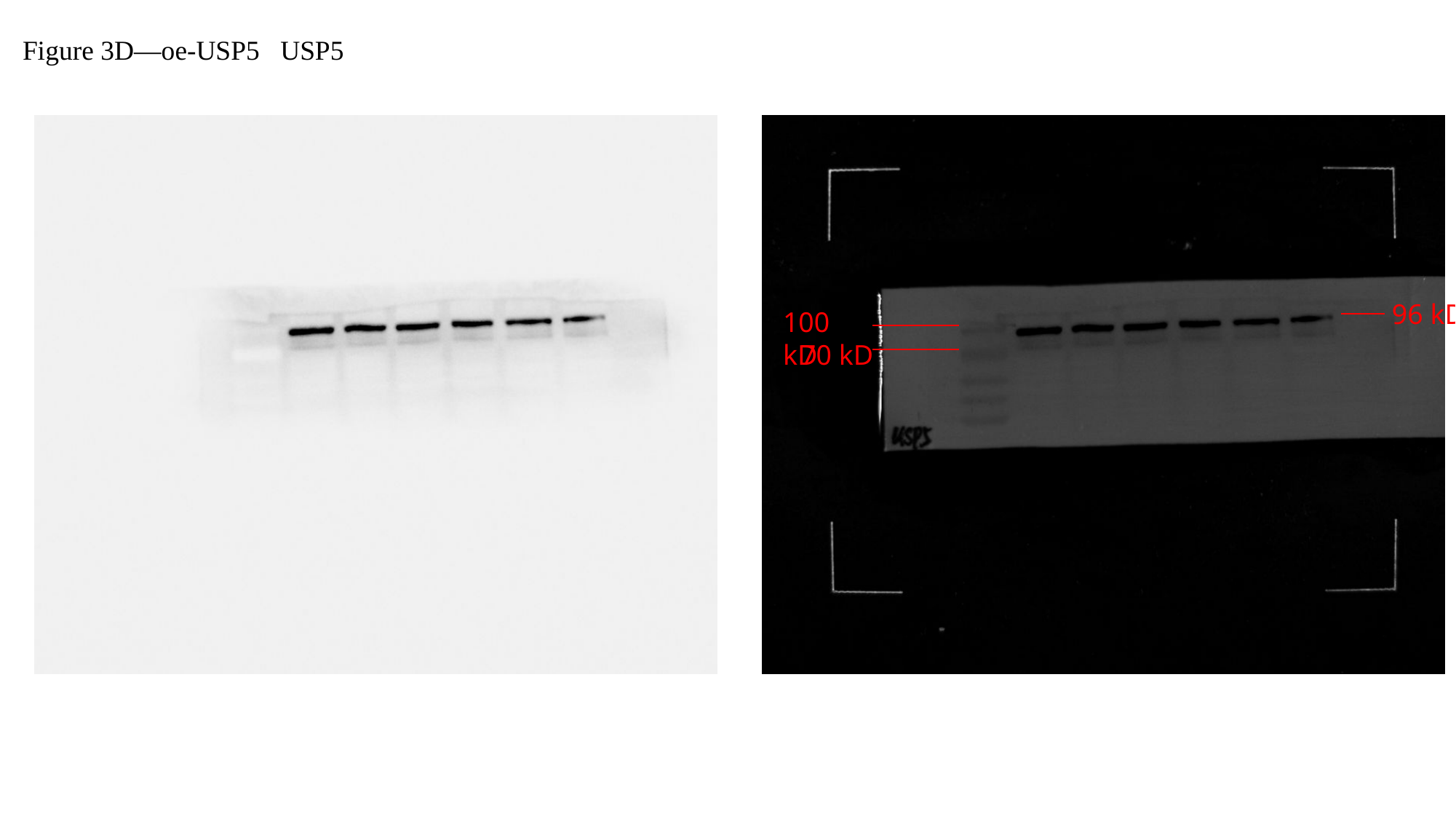

Figure 3D—oe-USP5 USP5
96 kD
100 kD
70 kD

## Slide 18
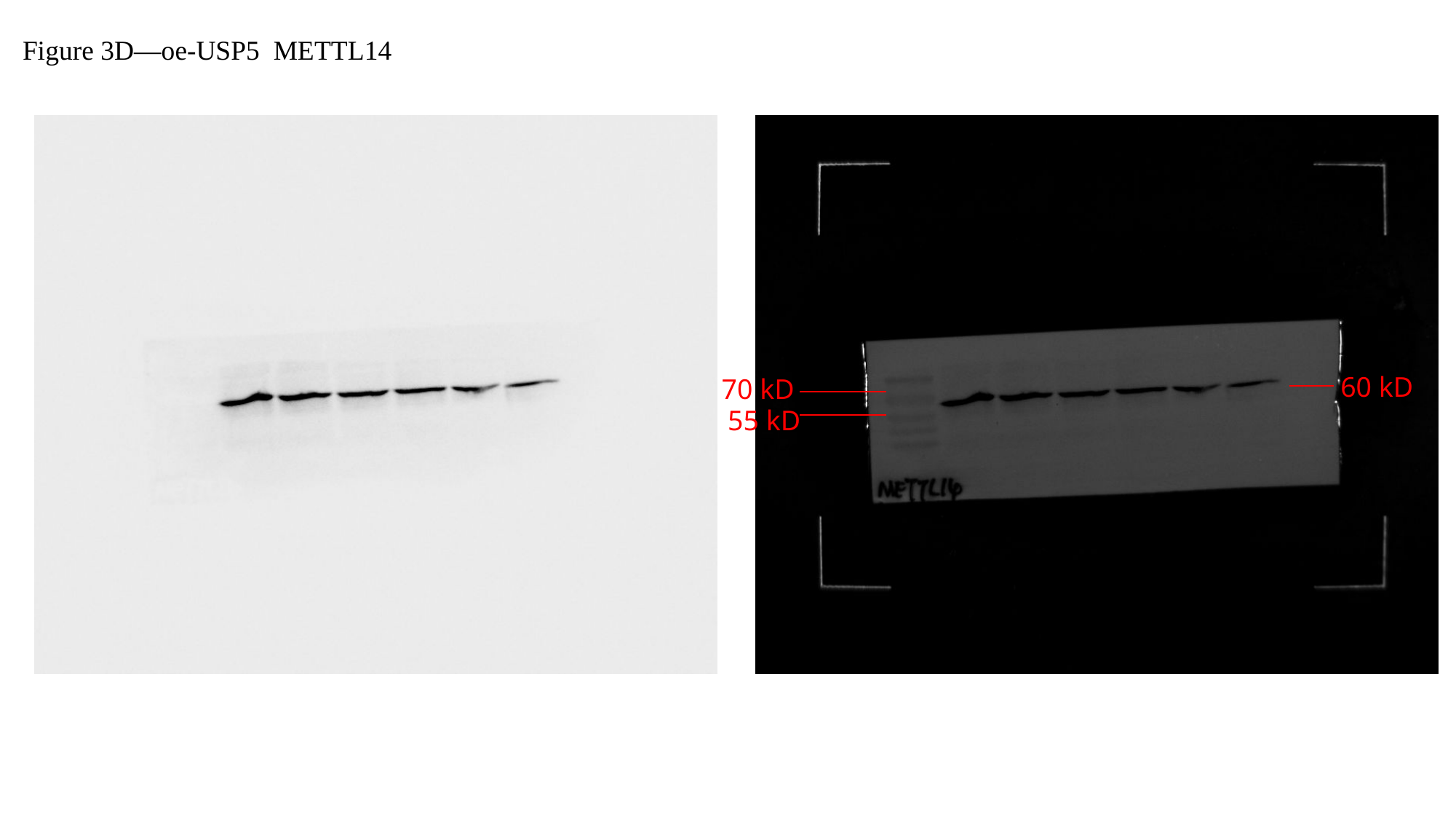

Figure 3D—oe-USP5 METTL14
60 kD
70 kD
55 kD

## Slide 19
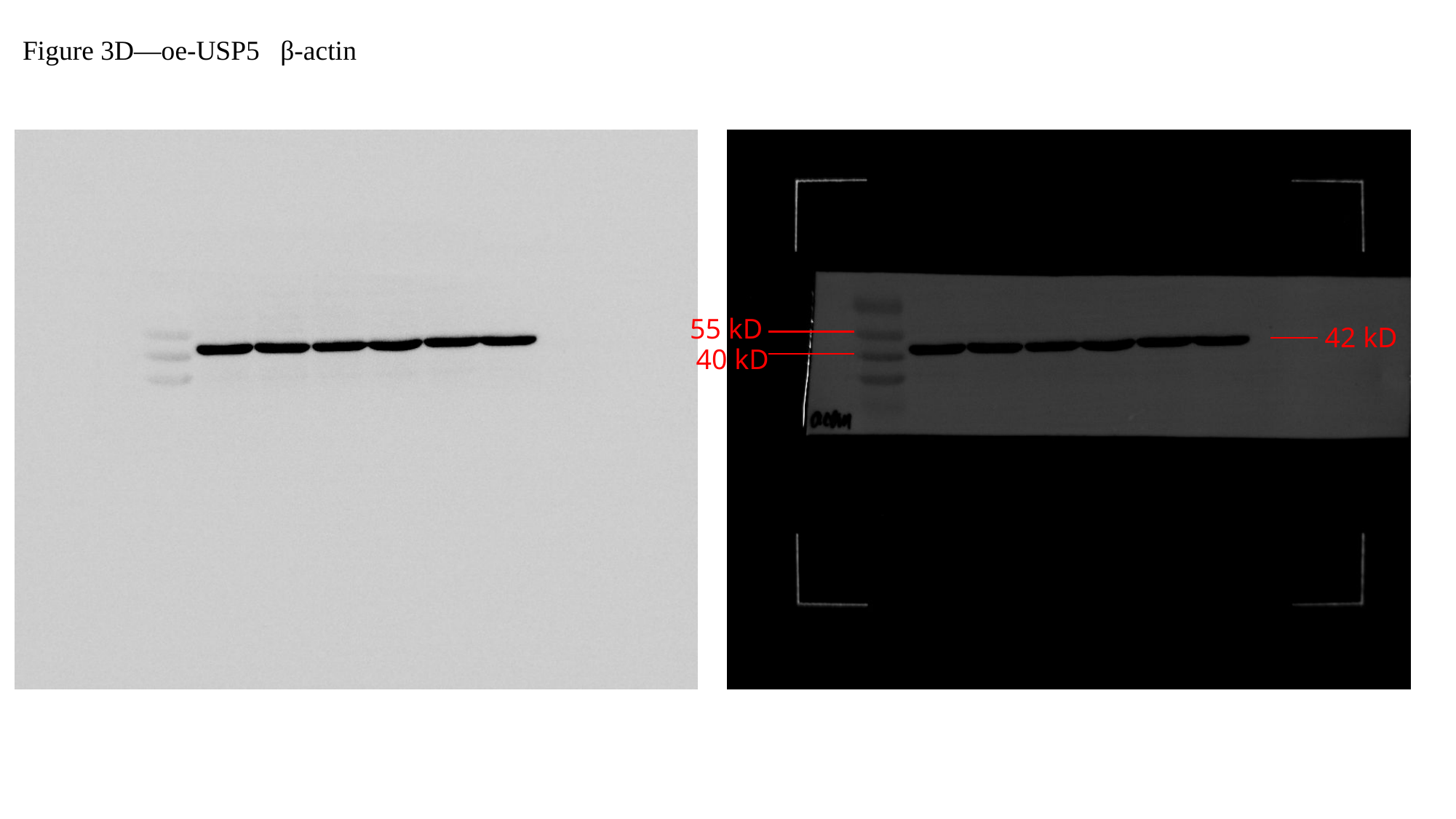

Figure 3D—oe-USP5 β-actin
55 kD
42 kD
40 kD

## Slide 20
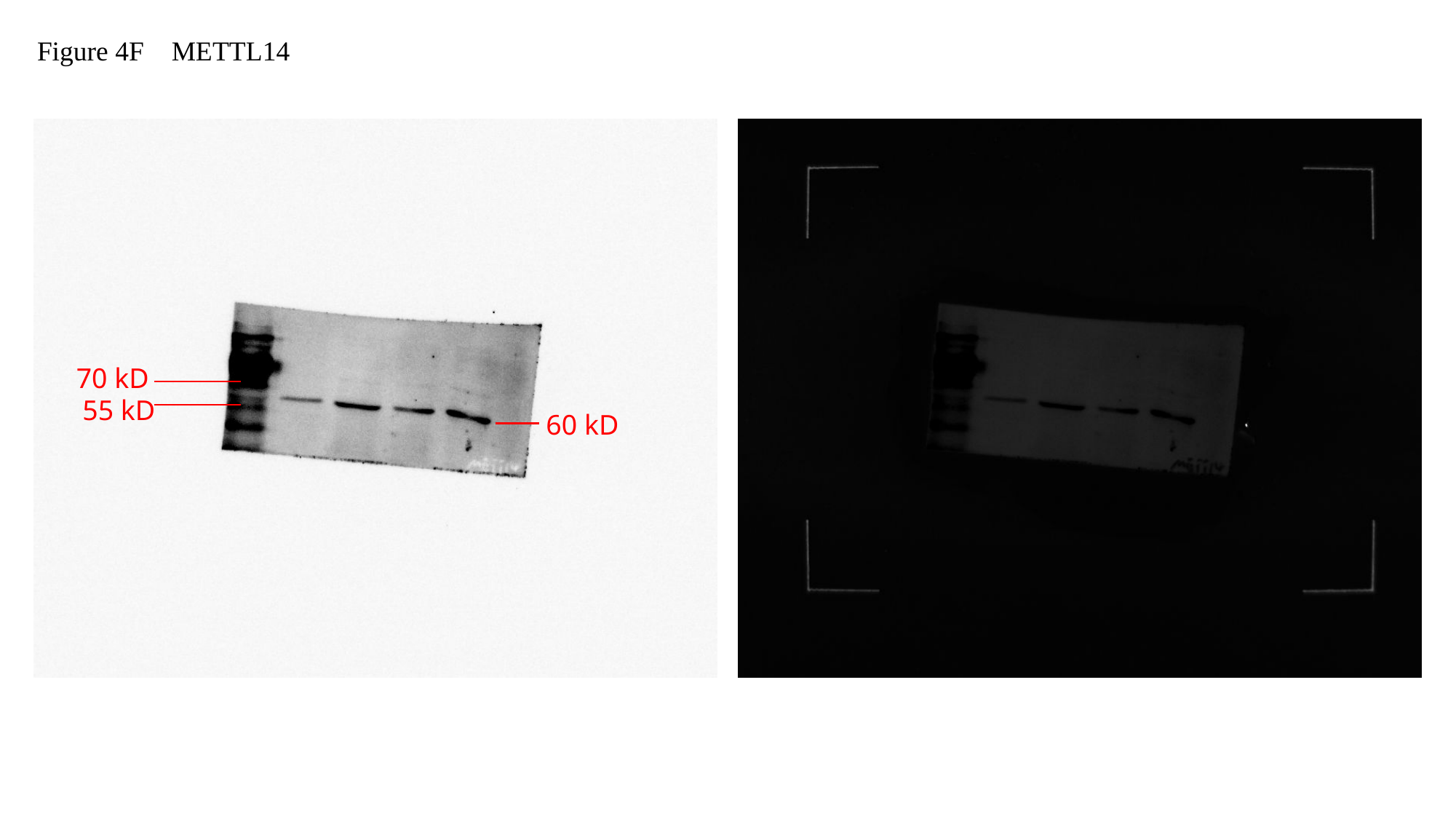

Figure 4F METTL14
70 kD
55 kD
60 kD

## Slide 21
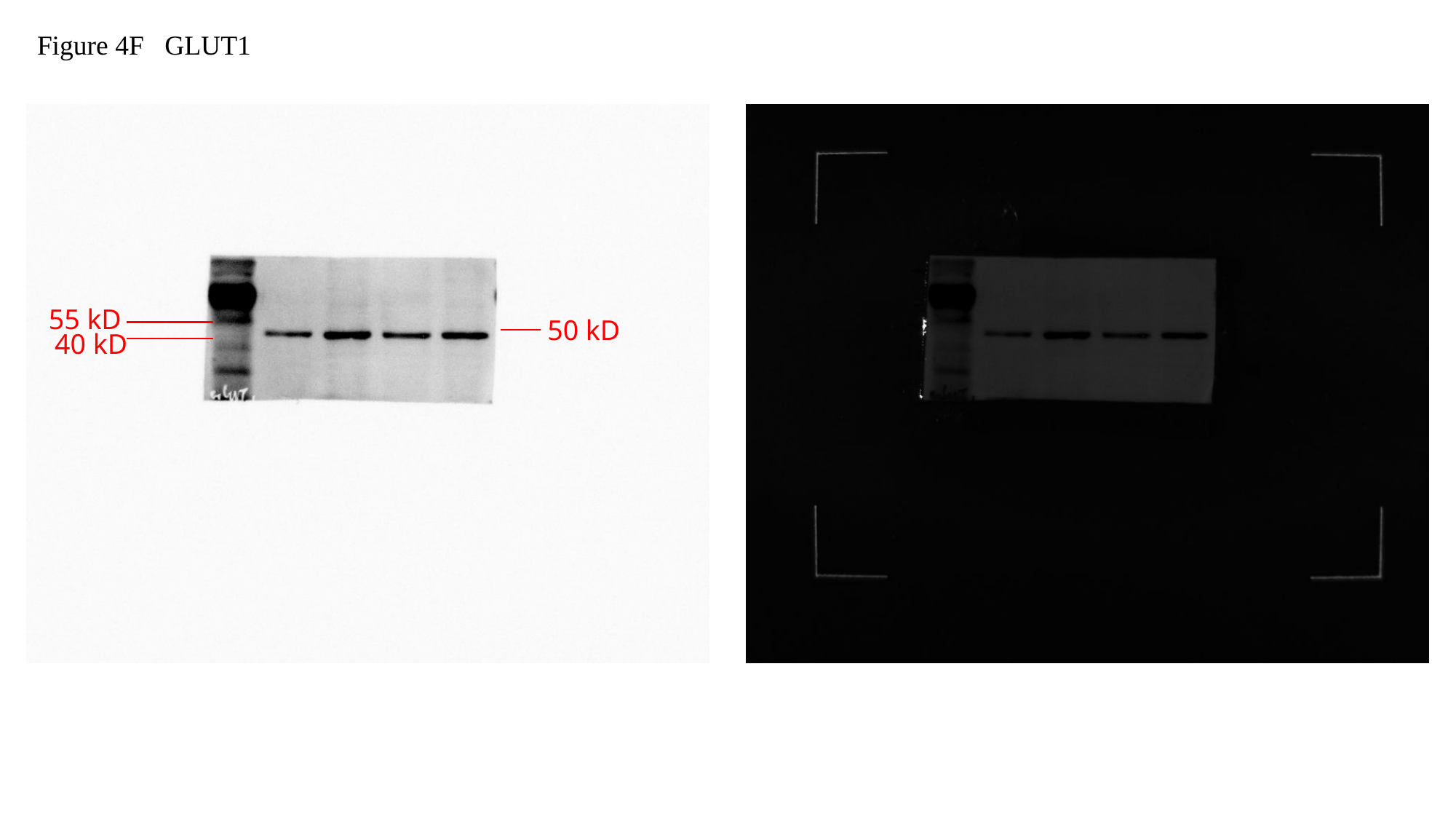

Figure 4F GLUT1
55 kD
50 kD
40 kD

## Slide 22
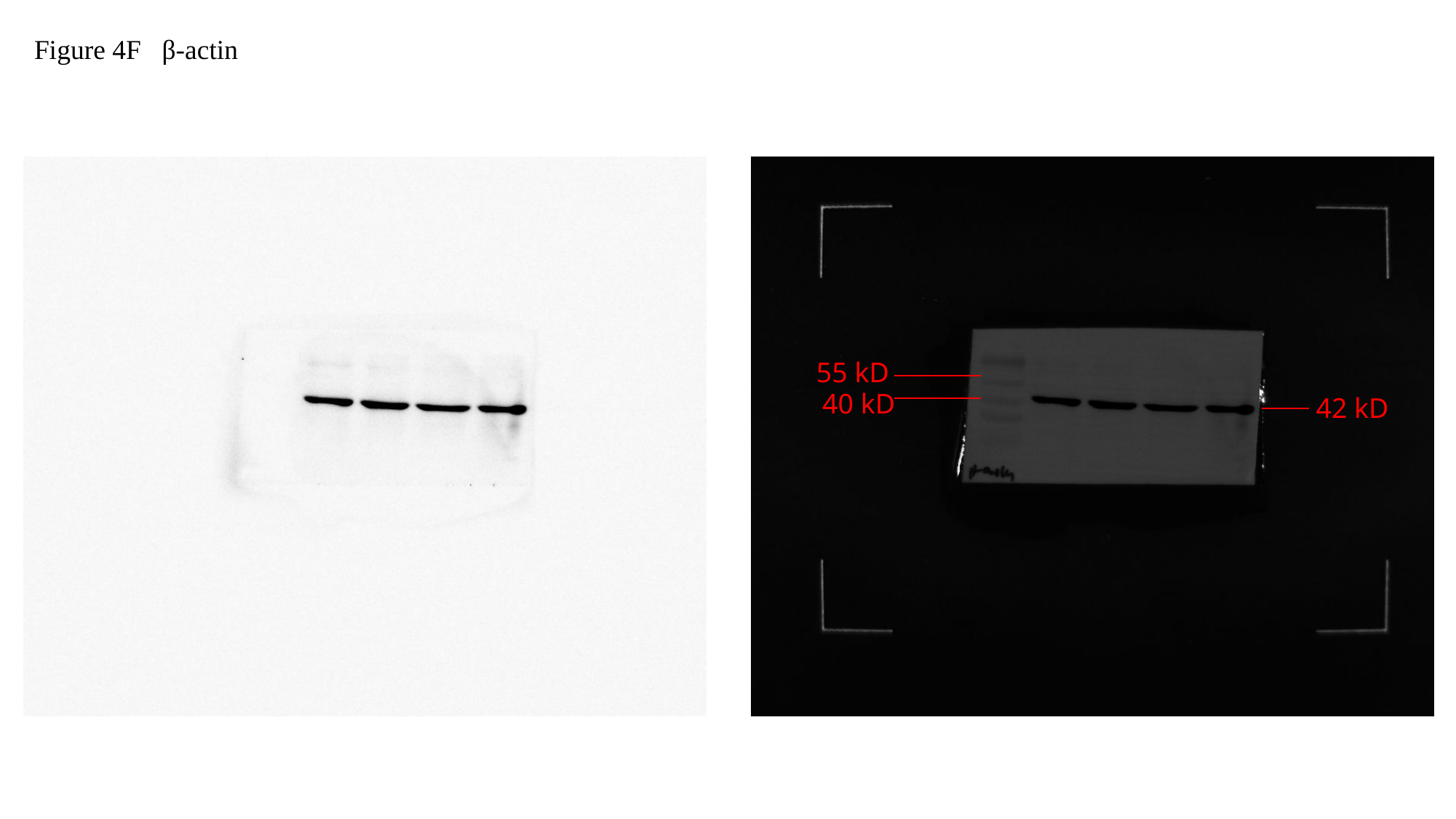

Figure 4F β-actin
55 kD
40 kD
42 kD

## Slide 23
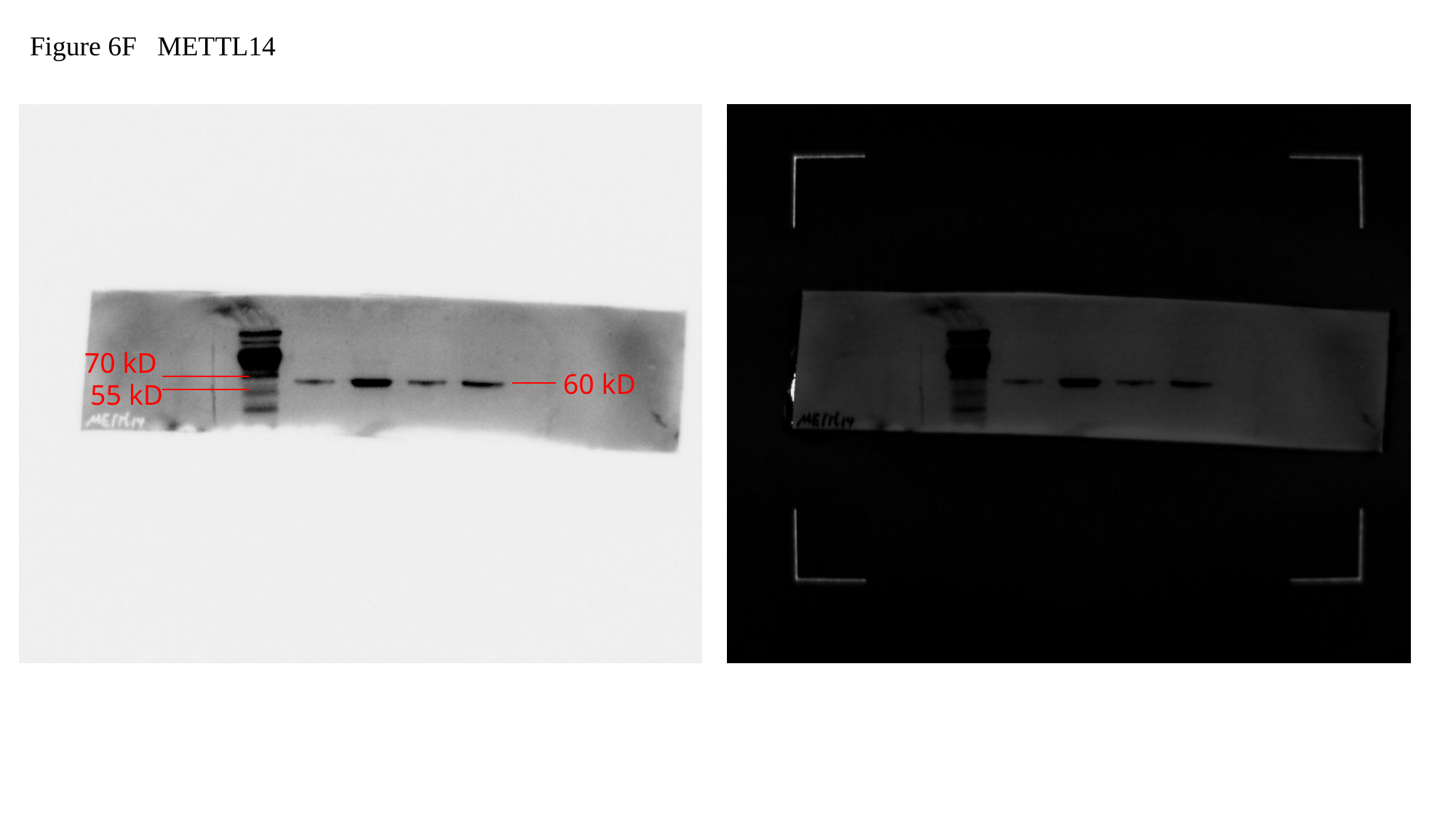

Figure 6F METTL14
70 kD
60 kD
55 kD

## Slide 24
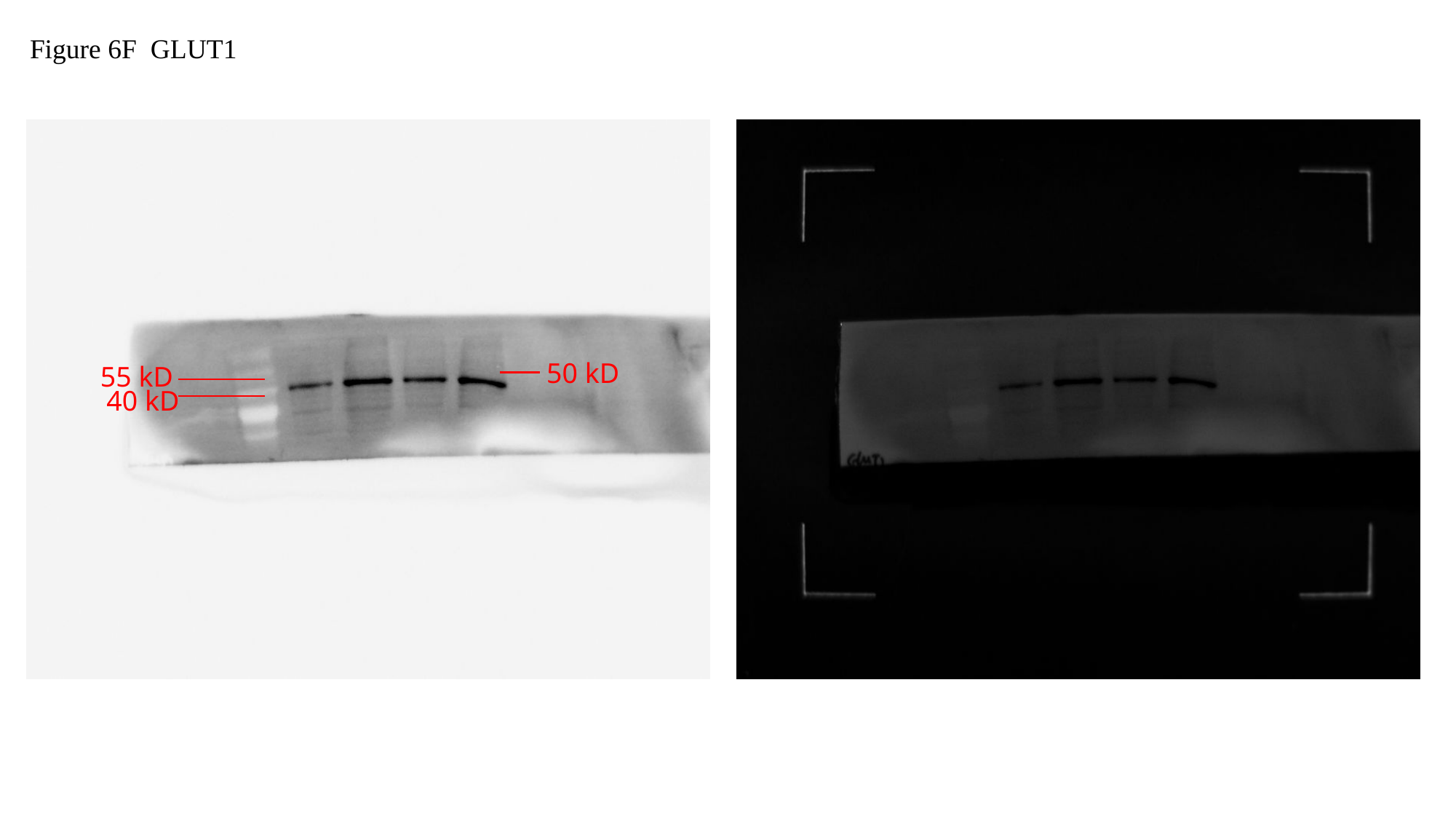

Figure 6F GLUT1
50 kD
55 kD
40 kD

## Slide 25
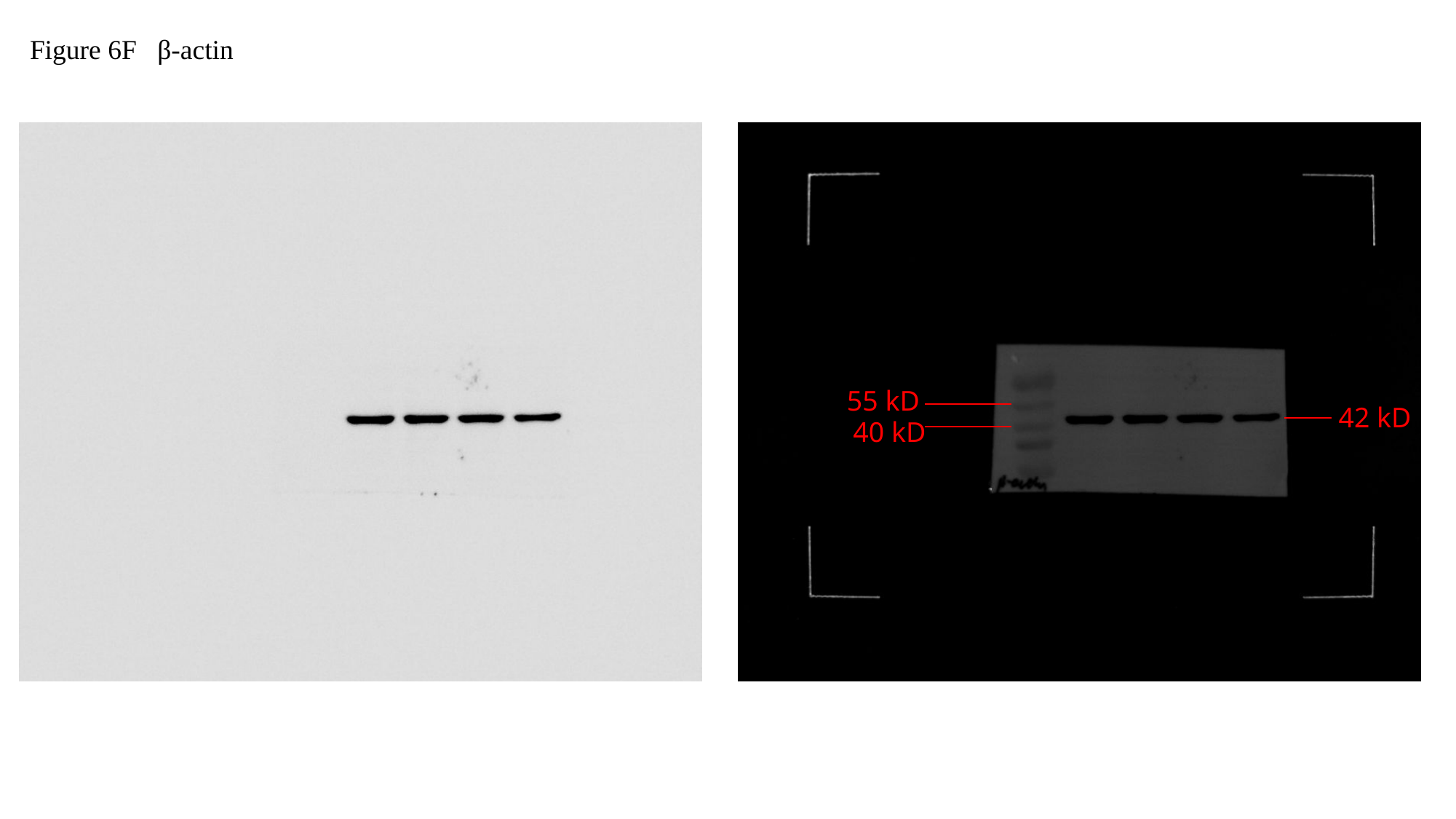

Figure 6F β-actin
55 kD
42 kD
40 kD

## Slide 26
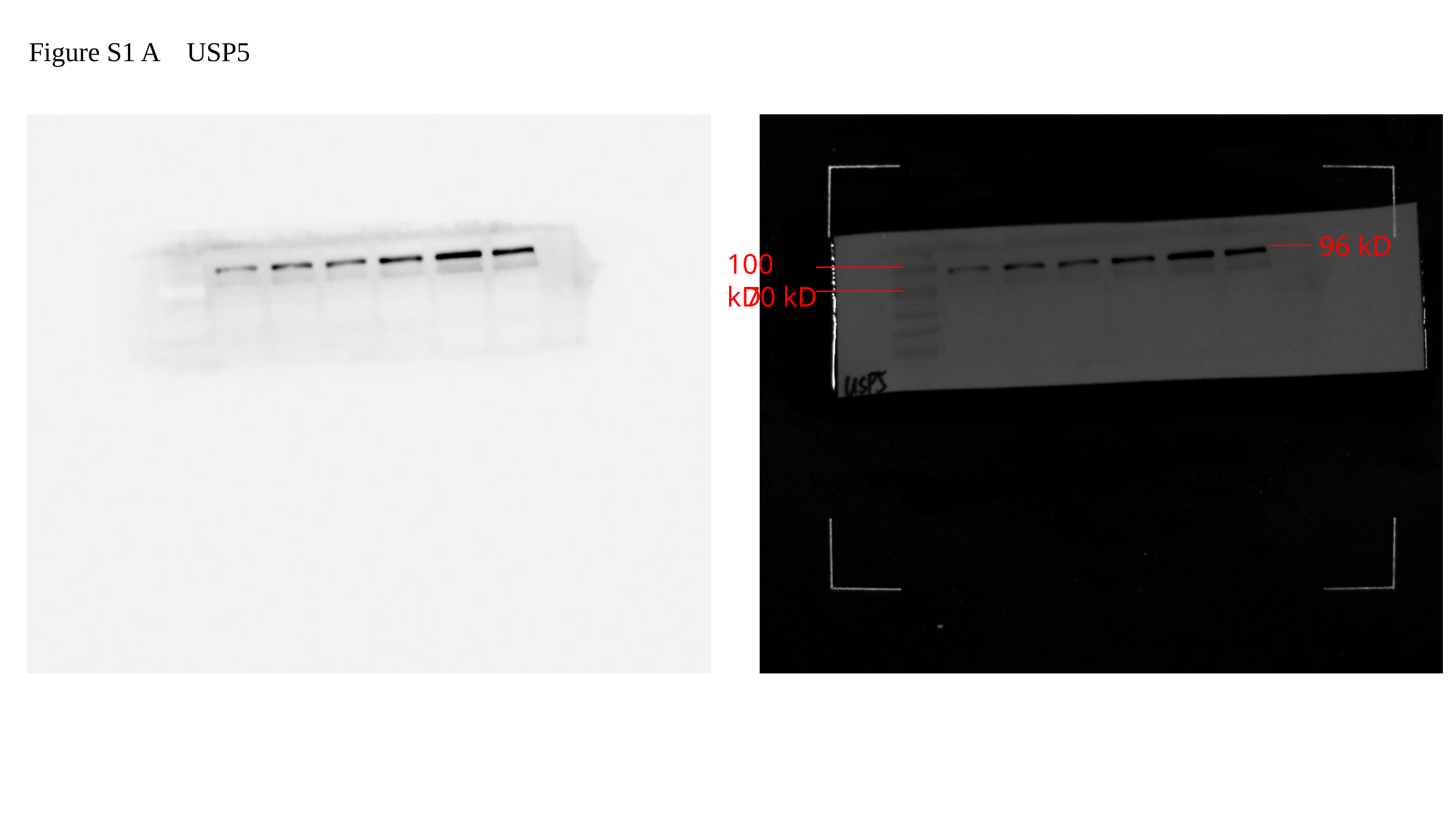

Figure S1 A USP5
96 kD
100 kD
70 kD

## Slide 27
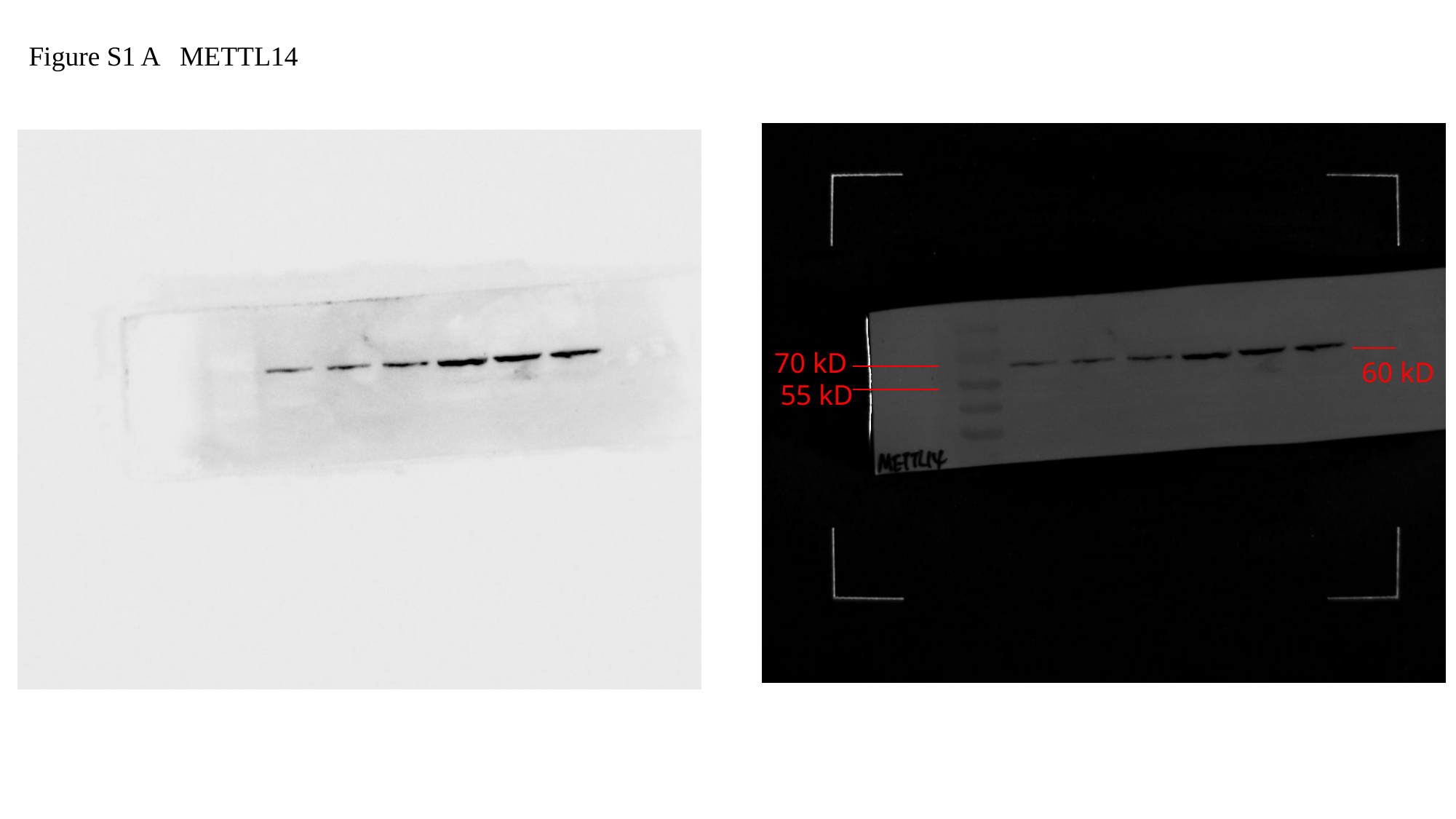

Figure S1 A METTL14
70 kD
60 kD
55 kD

## Slide 28
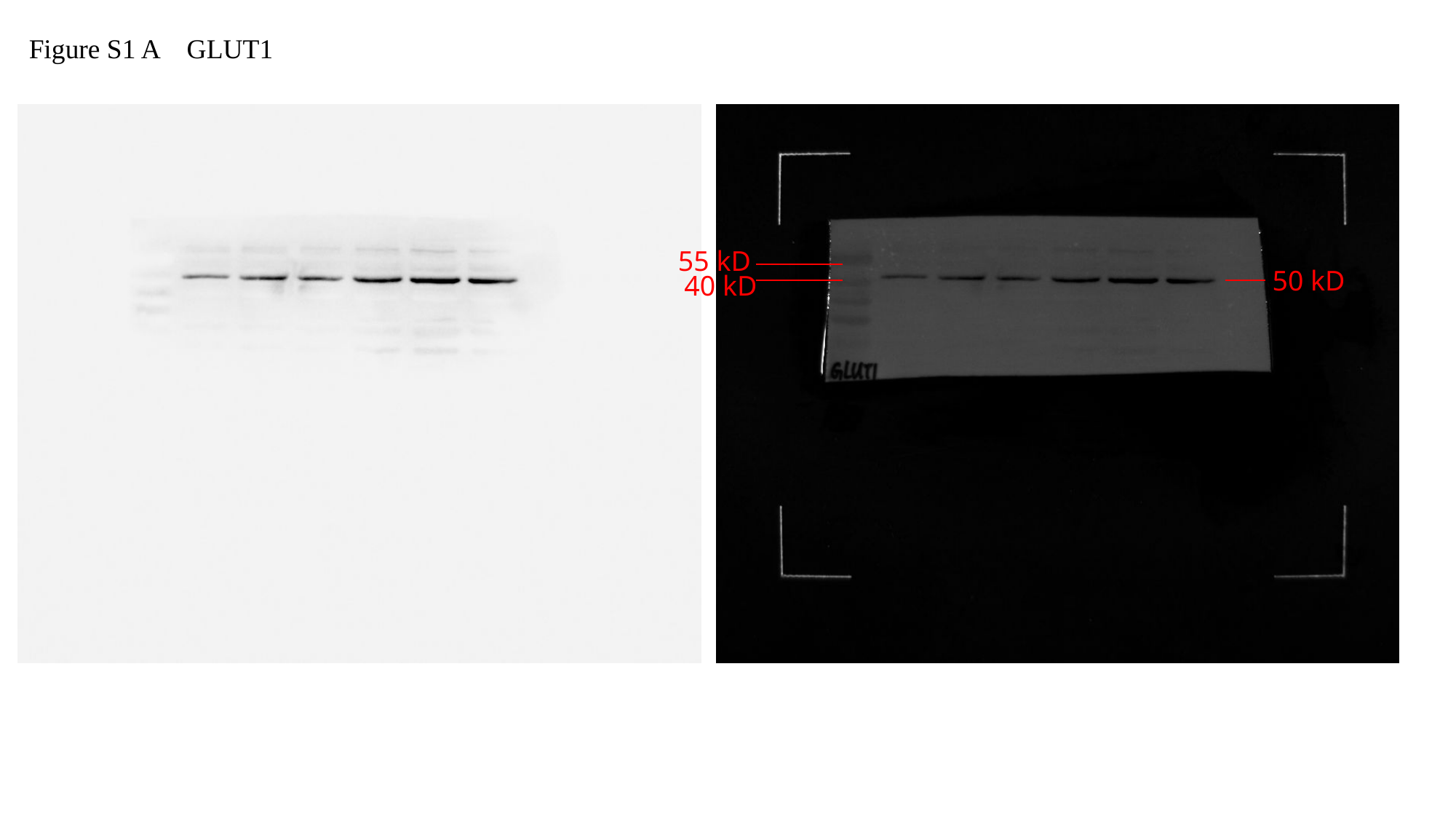

Figure S1 A GLUT1
55 kD
50 kD
40 kD

## Slide 29
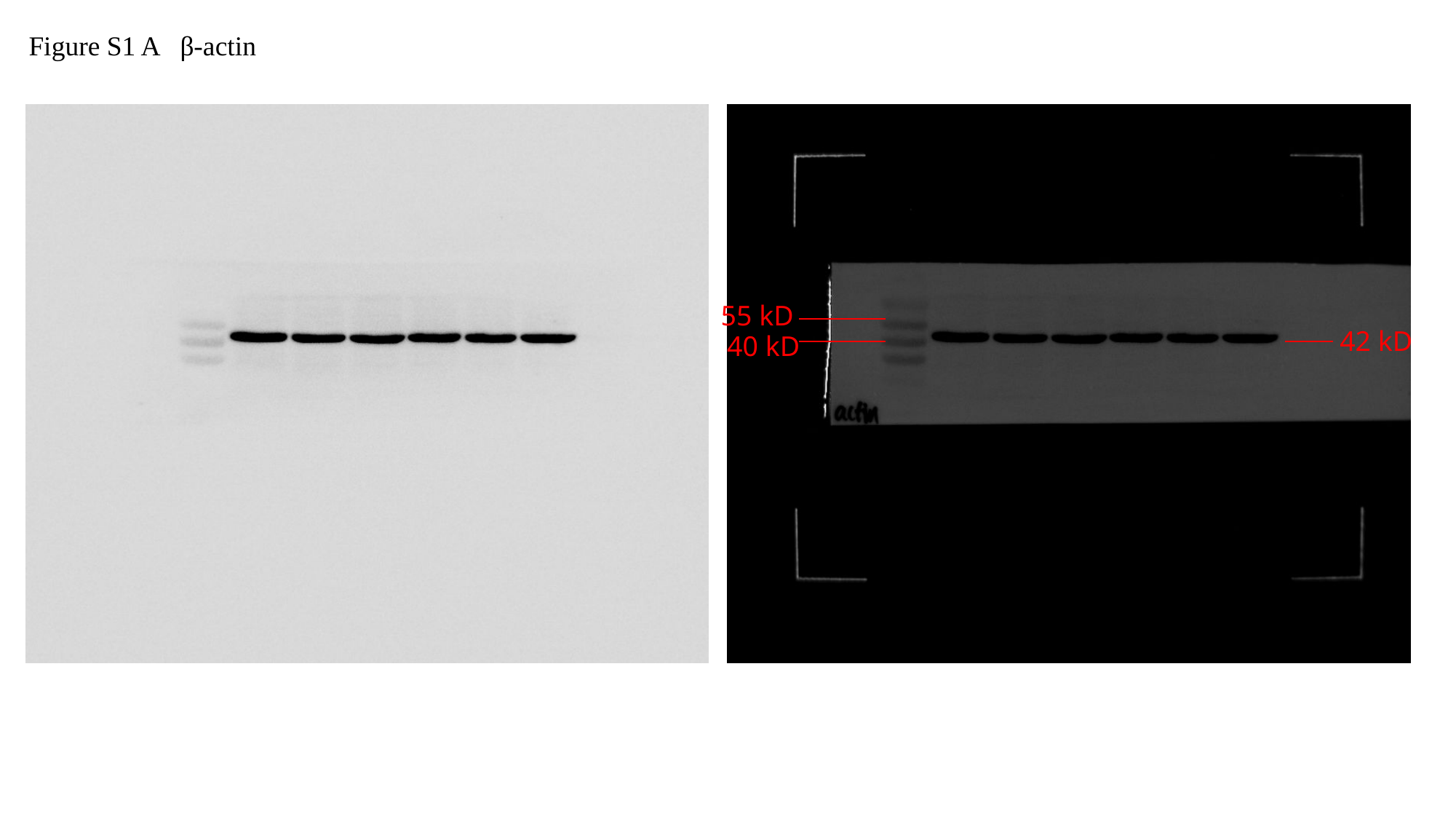

Figure S1 A β-actin
55 kD
42 kD
40 kD

## Slide 30
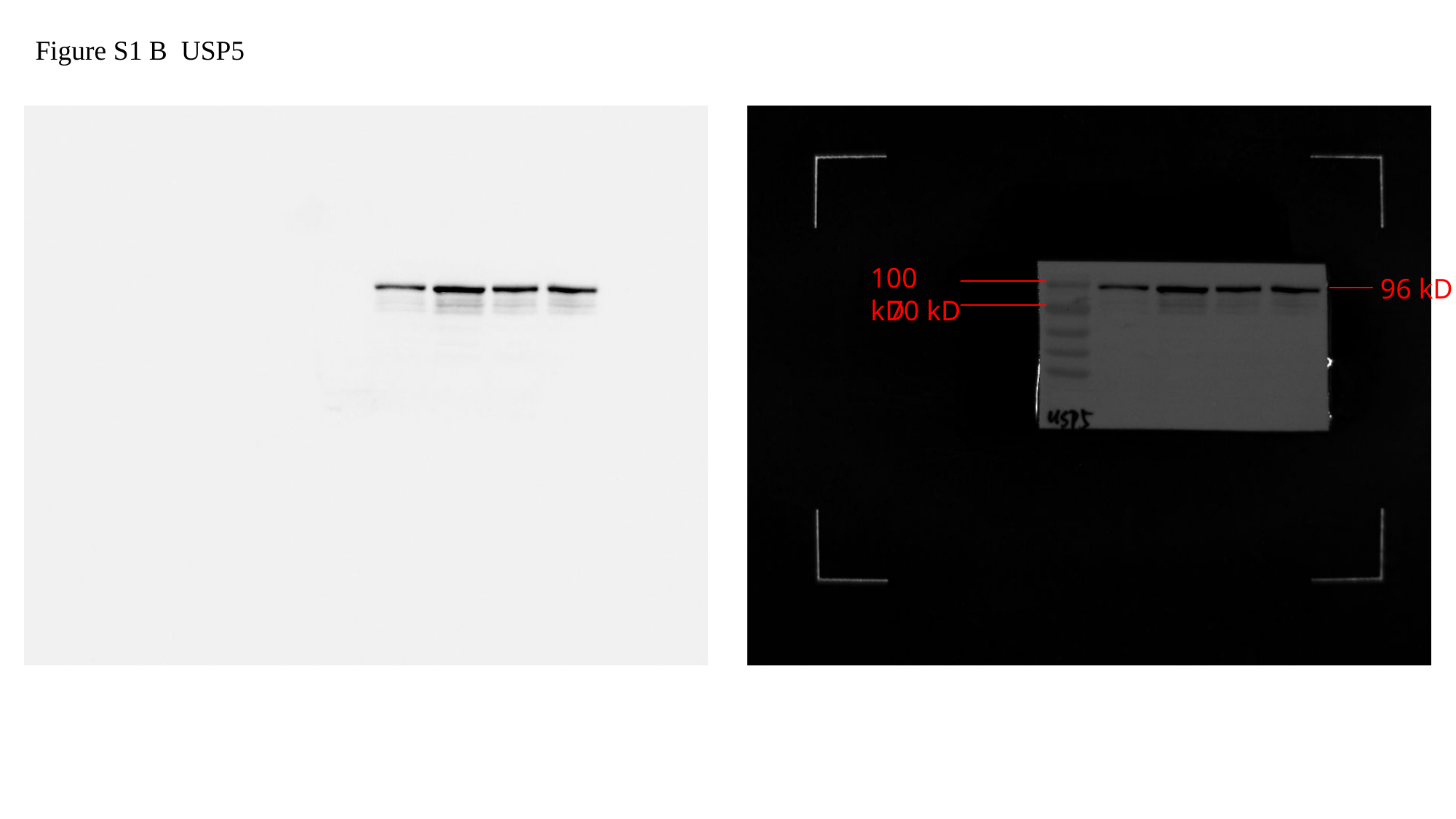

Figure S1 B USP5
100 kD
96 kD
70 kD

## Slide 31
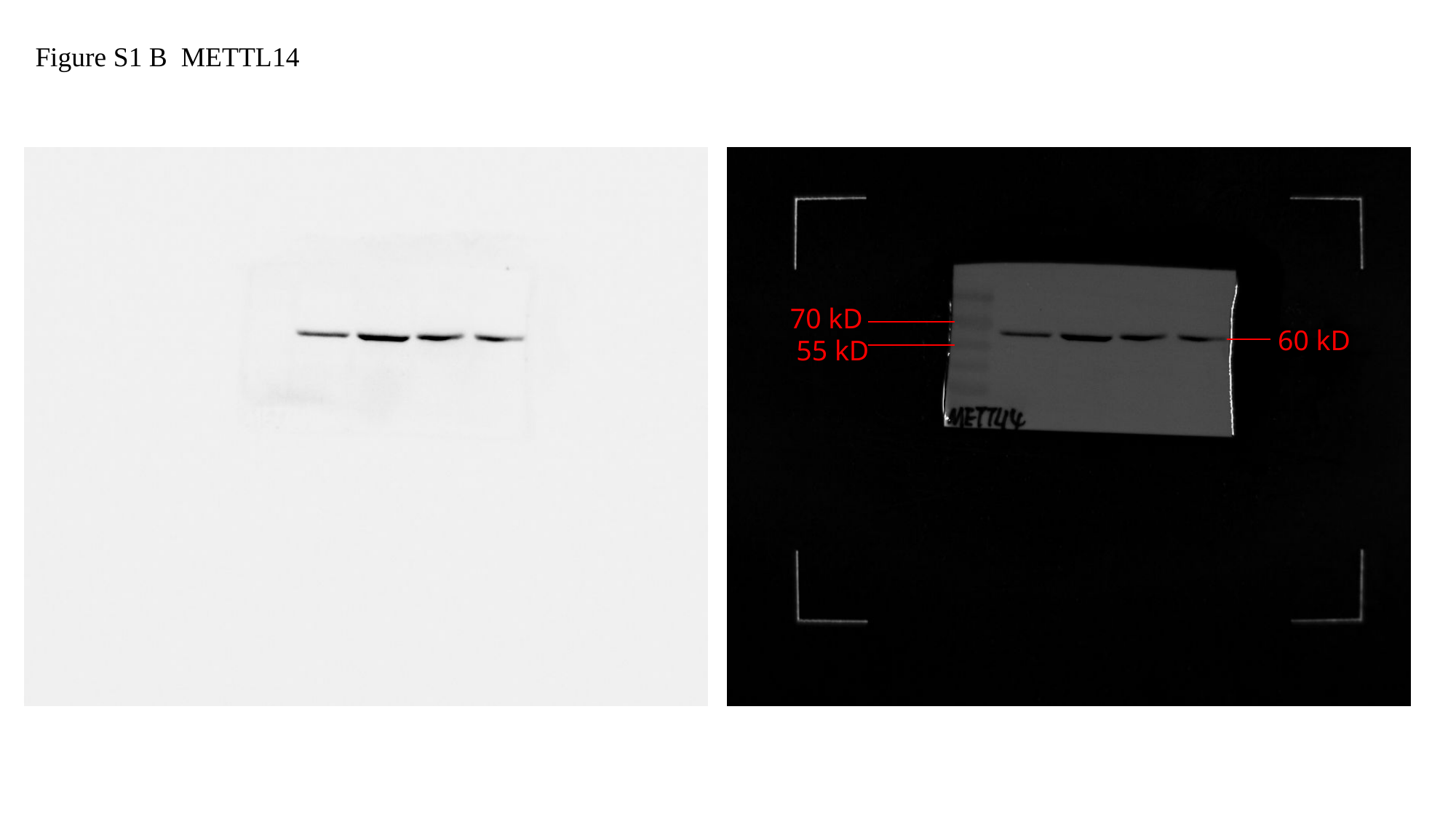

Figure S1 B METTL14
70 kD
60 kD
55 kD

## Slide 32
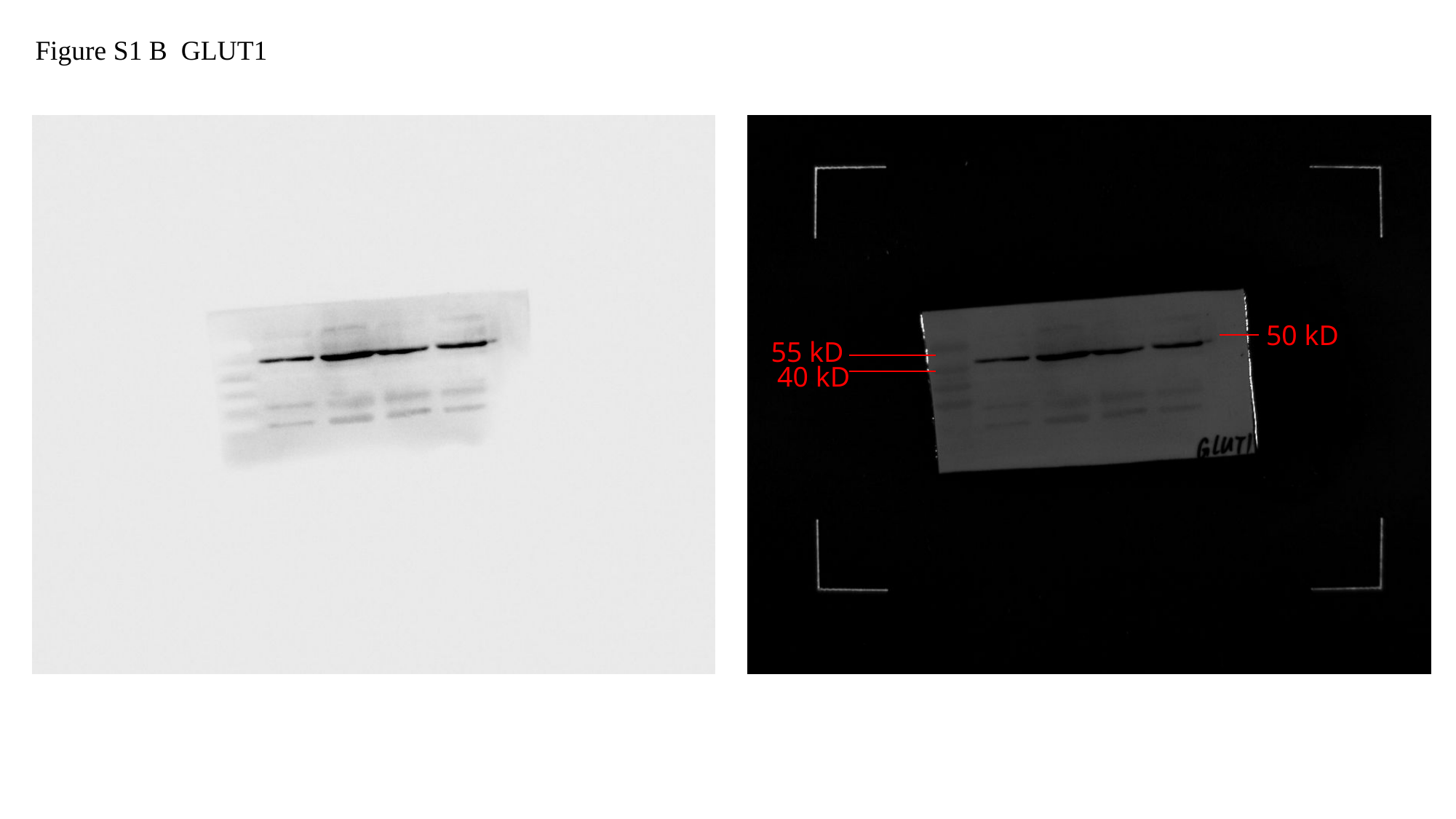

Figure S1 B GLUT1
50 kD
55 kD
40 kD

## Slide 33
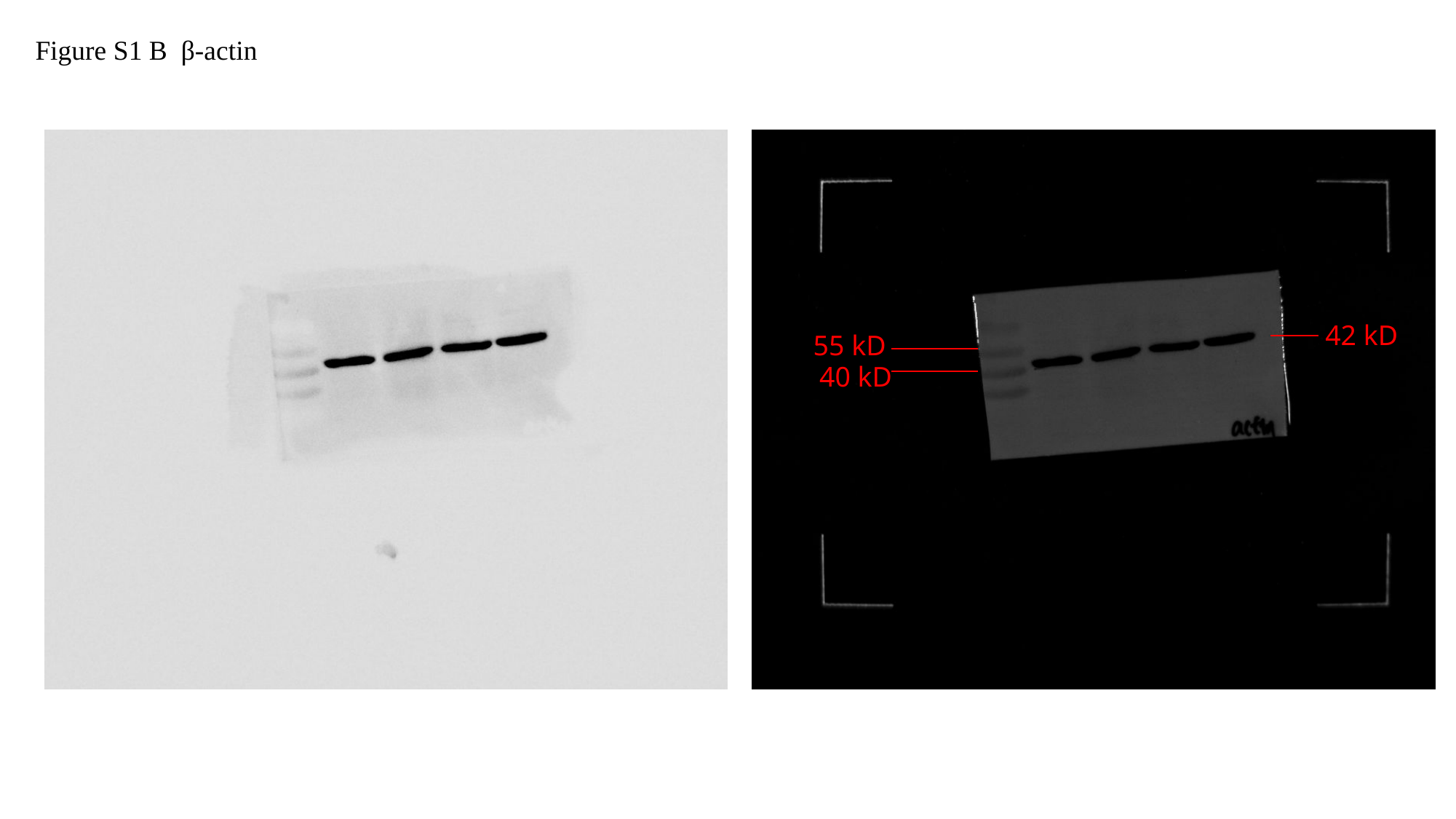

Figure S1 B β-actin
42 kD
55 kD
40 kD

## Slide 34
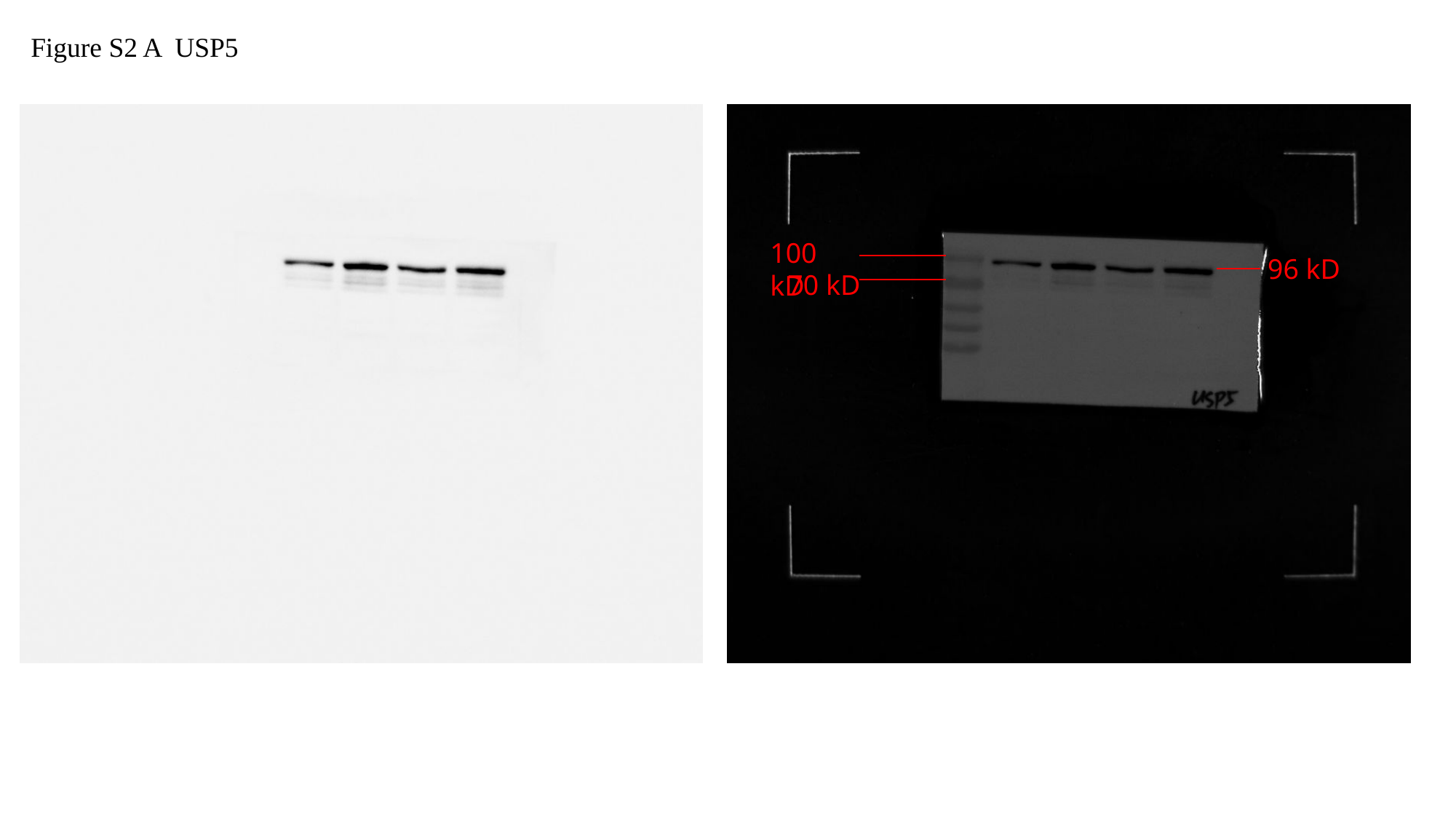

Figure S2 A USP5
100 kD
96 kD
70 kD

## Slide 35
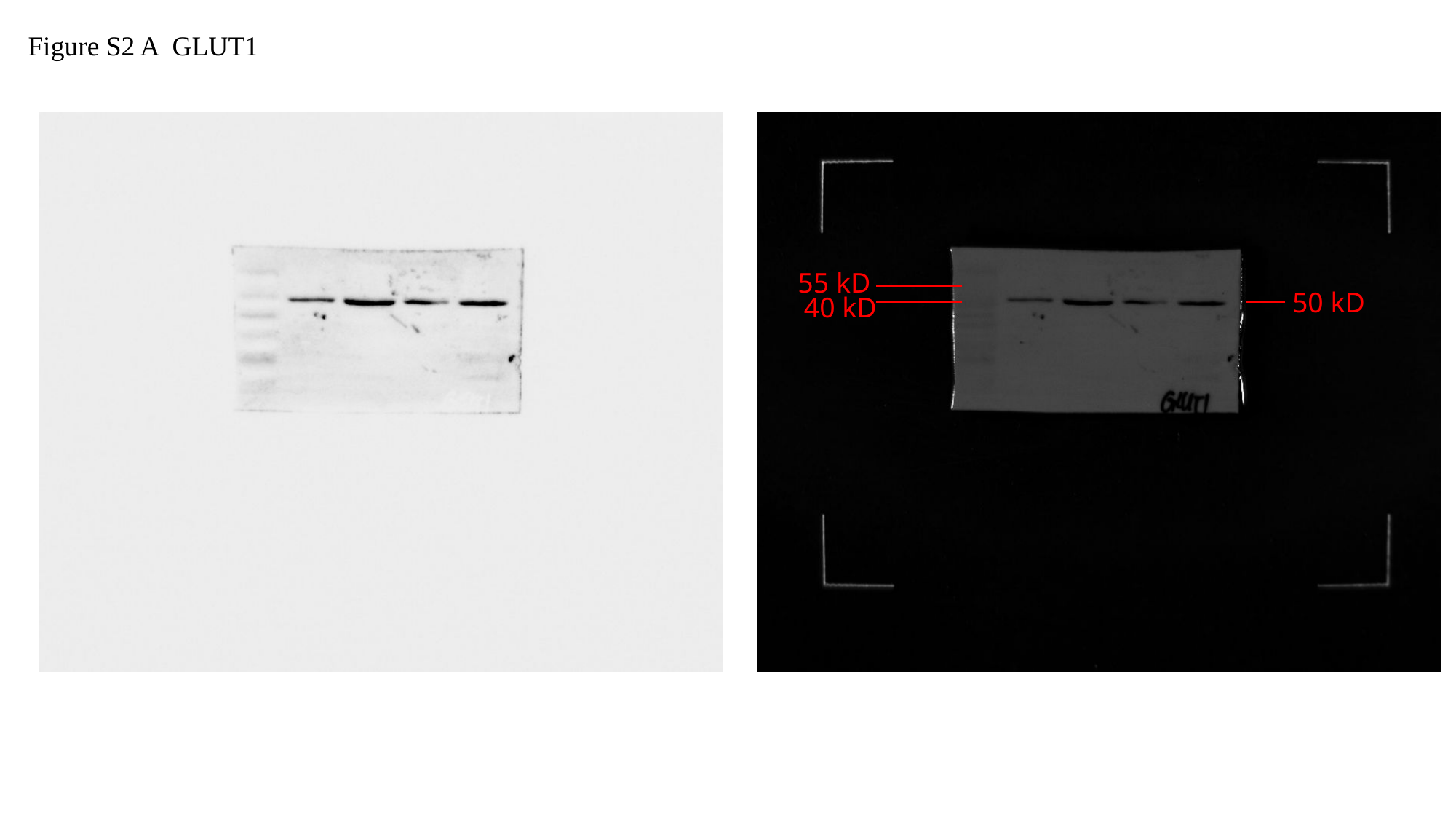

Figure S2 A GLUT1
55 kD
50 kD
40 kD

## Slide 36
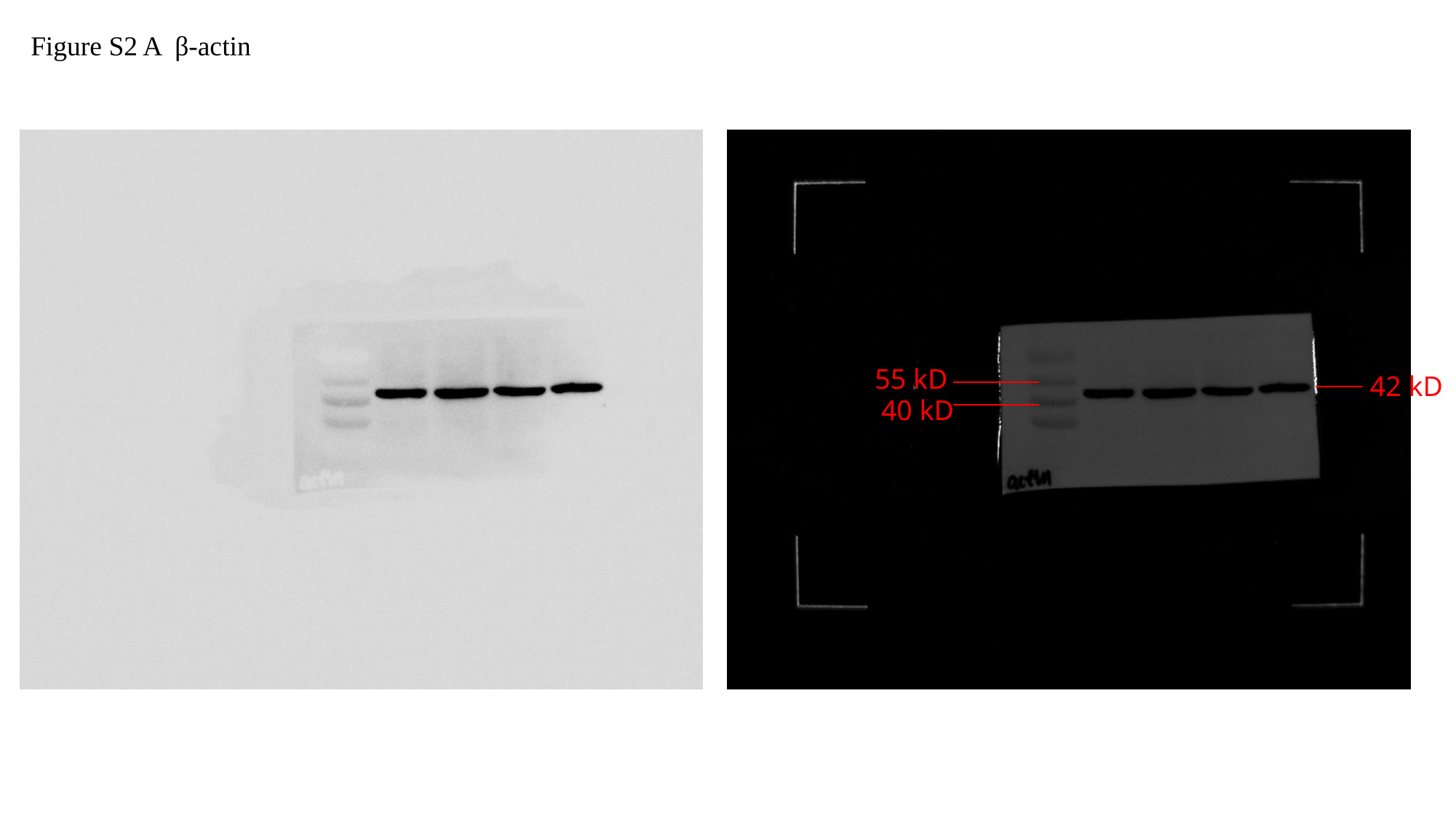

Figure S2 A β-actin
55 kD
42 kD
40 kD
